# MnO_2_–Co_3_O_4_@RGO hybrid nanocomposite-modified carbon paste electrode for sensitive electrochemical detection of hydrogen peroxide in food and edible-oil matrices

**DOI:** 10.1039/d6ra05902c

**Published:** 2026-10-12

**Authors:** Asmaa H. Ragab, Hend S. Mager, Rasha M. El-nashar, Rabeay Y. A. Hassan

**Affiliations:** a Biosensors Research Lab, Zewail City of Science and Technology 6th October City Giza 12578 Egypt ryounes@zewailcity.edu.eg +201129216152; b Applied Organic Chemistry Department, National Research Centre (NRC) Dokki Giza 12622 Egypt hendamer2000@yahoo.com; c Chemistry Department, Faculty of Science, Cairo University Giza 12613 Egypt

## Abstract

Repeated heating of cooking oils promotes lipid oxidation and the formation of reactive oxygen species (ROS) and other oxidation products, including hydrogen peroxide (H_2_O_2_). Excessive ROS generation can disrupt cellular redox homeostasis and contribute to oxidative stress, which is associated with cellular damage and biological processes implicated in carcinogenesis. Consequently, reliable methods for monitoring H_2_O_2_ in food and cooking oils are of interest for assessing oxidative changes in these matrices. In this work, a non-enzymatic electrochemical sensor was developed for the rapid and sensitive direct determination of H_2_O_2_ in food and oil samples. A carbon paste electrode was modified with a hybrid nanocomposite comprising MnO_2_ and Co_3_O_4_ nanostructures supported on reduced graphene oxide (RGO) nanosheets to construct an efficient electrochemical sensing interface. The structural, morphological, and electrochemical characteristics of the synthesized materials and modified electrodes were investigated using transmission electron microscopy (TEM), Fourier-transform infrared (FTIR) spectroscopy, X-ray diffraction (XRD), electrochemical impedance spectroscopy (EIS), and cyclic voltammetry (CV). Following optimization, the MnO_2_–Co_3_O_4_@RGO-modified electrode exhibited enhanced electrocatalytic activity toward the H_2_O_2_ oxidation and was evaluated by chronoamperometry (CA), achieving a detection limit of 0.03 µM and a wide dynamic linear range of 0.1–1800 µM. Furthermore, the sensor exhibited good selectivity toward H_2_O_2_ in the presence of common interfering species and high sensor surface stability. Thus, its practical applicability was evaluated using H_2_O_2_-spiked burger, salami, and cheese samples and repeatedly heated palm-oil samples, with satisfactory recoveries obtained. Accordingly, the newly developed MnO_2_–Co_3_O_4_@RGO-modified electrode provides a simple, cost-effective, and efficient platform for non-enzymatic H_2_O_2_ determination, with potential applications in monitoring oxidative changes in cooking oils and selected food matrices.

## Abstract 2

Synthesis of MnO_2_–Co_3_O_4_@RGO nanocomposite and its incorporation into a carbon paste electrode for electrochemical H_2_O_2_ detection, with application in food-quality monitoring.

## Graphical abstract

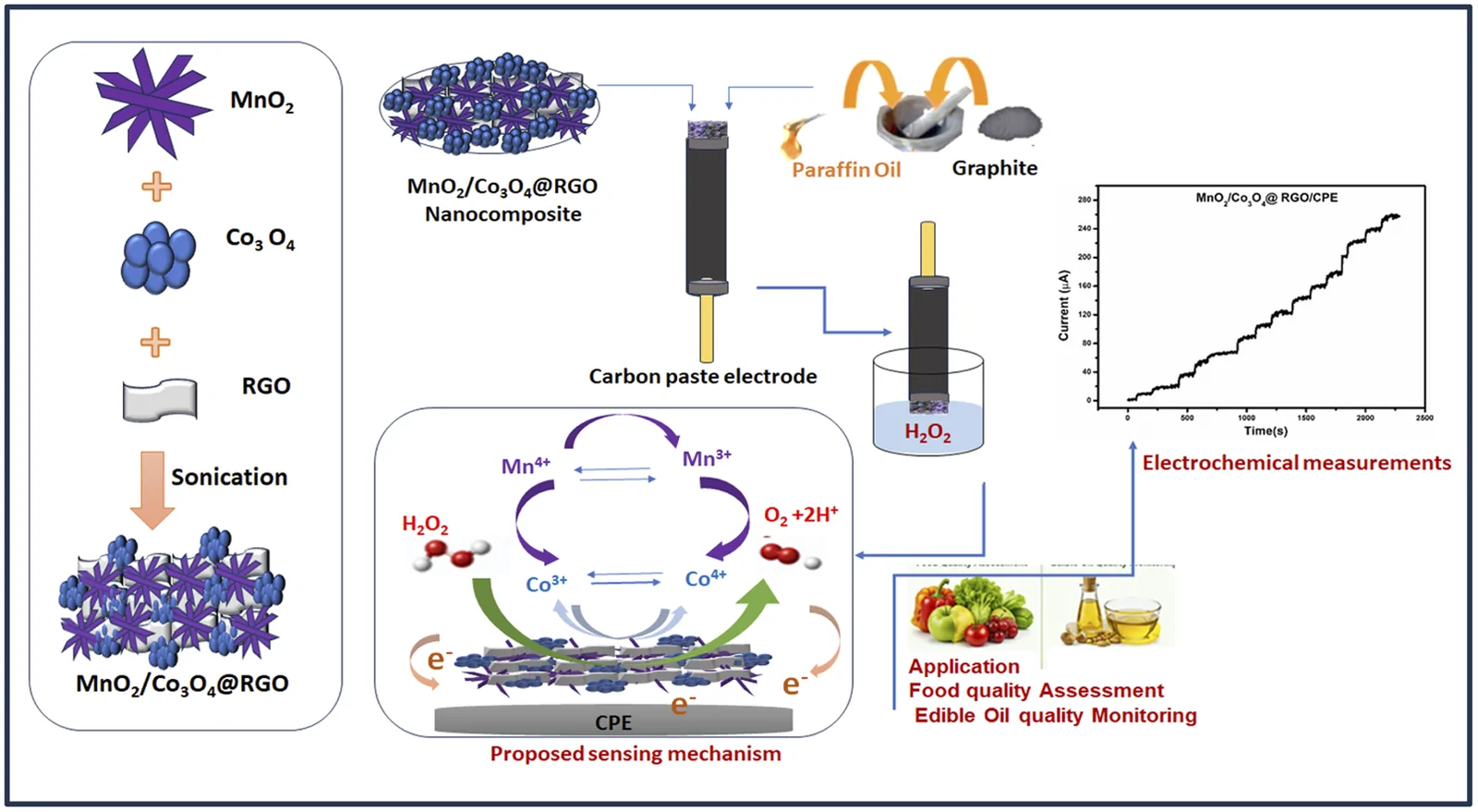

## 1. Introduction

Cancer diseases remain one of the major global health challenges. Despite extensive research into their diagnosis and treatment, effective management of many cancer types remains challenging, and a definitive cure is not available for all forms of the disease. Because prevention can substantially reduce cancer risk, minimizing exposure to established carcinogenic substances represents an important and practical strategy for cancer prevention.^1^ In this context, commonly used cooking oils may become a potential source of harmful oxidation products, particularly when subjected to prolonged or repeated heating, which may contribute to processes associated with gastrointestinal cancer risk.^2,3^ Fried foods are a substantial component of modern diets, while repeated heating of frying oils promotes lipid oxidation and generates reactive oxygen species, including H_2_O_2_.^3,4^ These compounds are collectively known as reactive oxygen species (ROS),^5^ which play important roles in physiological processes, including enzymatic activity, cellular signaling, mitochondrial electron transport, gene regulation, and inflammatory responses.

Under normal conditions, endogenous antioxidant systems regulate ROS levels and maintain redox balance. Key enzymes, including glutathione peroxidase, catalase, and superoxide dismutase, provide the primary enzymatic defence, while non-enzymatic antioxidants further neutralize reactive species.^6^ Maintaining a balance between ROS generation and antioxidant capacity is essential for cellular homeostasis. When excessive ROS production overwhelms the antioxidant defence system, oxidative stress occurs, leading to oxidative damage such as lipid peroxidation and protein damage.^7^ In particular, oxidative stress may disrupt cellular signalling pathways or induce DNA damage, potentially activating oncogenes and contributing to cancer development.^8^ Because ROS can act as carcinogenic agents, and repeated heating of cooking oils promotes their formation, frequent consumption of such oils may upset the body's internal balance and initiate carcinogenic processes. Furthermore, hydrogen peroxide is a widely utilized chemical compound with significant applications across diverse sectors, including pharmaceuticals, chemical manufacturing, fuel cells, paper industries, textile, and food.^9,10^ Owing to its extensive use, the accurate detection and monitoring of H_2_O_2_ have become increasingly important. Given these considerations, the development of reliable methods for H_2_O_2_ detection is of great importance in both environmental and biomedical fields. Conventional analytical techniques, including chemiluminescence,^11^ titration,^12^ colorimetric analysis,^13^ UV-visible spectrophotometry,^14^ chromatography,^15^ and fluorescence-based methods,^16^ have been widely employed for this purpose. However, these approaches often involve expensive instrumentation, labor-intensive procedures, and time-consuming sample preparation, limiting their practical applicability. As a result, there is still a lack of dependable tools for real-time evaluation and rapid determination of peroxides. This highlights the need for a portable, point-of-care device capable of providing rapid and on-site assessment of cooking oil quality.

Electrochemical sensing techniques have emerged as attractive alternatives due to their advantages, including the rapid response, high sensitivity, cost-effectiveness, and suitability for on-site analysis.^17–19^ Among these, voltammetric and chronoamperometric techniques are particularly important for the sensitive and selective accurate determination of electrochemically active small and macromolecular analytes. Their versatility, relatively simple instrumentation, and rapid response make them promising approaches for near-real-time monitoring with minimal operational complexity.^20^ Enzyme-based electrochemical sensors have demonstrated excellent selectivity and sensitivity;^21,22^ however, their practical use is restricted by issues such as enzyme instability, high production cost, and complicated immobilization processes.

To overcome these limitations, more attention has been focused on non-enzymatic electrochemical sensors that utilize nanostructured materials as catalytic elements. These nanostructured electrodes enabled several benefits, including improved stability, faster response, lower cost, and independence from environmental conditions.^23,24^ Therefore, development of nanostructured electrode materials with high electrical conductivity, enhanced electrocatalytic activity, and long-term stability remains a major focus in this field. Their high specific surface area, abundant accessible electroactive sites, and efficient electron-transfer characteristics will support targeting analyte(s) accumulation and interfacial charge transfer, thereby improving the overall sensitivity, selectivity, and the analytical performance of designed electrochemical sensors.^25^

For instance, a previously reported Nafion/exfoliated graphene oxide-Co_3_O_4_-modified electrode achieved a detection limit of approximately 0.3 µM, a response time of approximately 4 s, and a linear detection range spanning several orders of magnitudee.^26^ Additionally, Co_3_O_4_/RGO-electrodes have been reported for non-enzymatic H_2_O_2_ detection with a linear range of 0.1–19.5 mM, a sensitivity of 0.2247 mA mM^−1^ cm^−2^, and a detection limit of 10 µM.^27^ A MnO–Mn_3_O_4_@RGO electrode exhibited a wide linear range of 0.004–17 mM and a low detection limit of approximately 0.1 µM, demonstrating the contribution of conductive graphene and mixed metal-oxide structures to H_2_O_2_ electrocatalysis.^28^ A Co_3_O_4_–Ni nanoparticle composite showed a linear range of 0.5–89 µM and a detection limit of 0.23 µM for H_2_O_2_.^29^ More recently, hierarchical Co_3_O_4_ has been reported for H_2_O_2_ detection over 4–3160 µM, with a detection limit of 1.6 µM and a normalized sensitivity of 0.284 µA µM^−1^ cm^−2^.^30^ A Co_3_O_4_-based electrochemical sensor has also been reported for hydroperoxide determination in edible oils, with a rapid response of <20 s and an H_2_O_2_ detection limit of approximately 10^−7^ M.^31^

In particular, manganese dioxide (MnO_2_) and cobalt oxide (Co_3_O_4_)^32^ exhibit favorable redox and electrocatalytic properties that can facilitate electron transfer during the electrochemical reaction of H_2_O_2_.^33,34^ When combined with reduced graphene oxide (RGO),^35^ which provides a highly conductive framework and a large accessible surface area, the resulting nanocomposite can benefit from synergistic interactions among its components. These complementary properties facilitate charge transport, increase the accessibility of electroactive sites, and enhance the overall electrocatalytic performance of the sensing interface.

The electrocatalytic response toward H_2_O_2_ at MnO_2_–Co_3_O_4_@RGO-modified electrodes can be attributed to the redox-active manganese and cobalt oxide sites, which facilitate the interfacial electron-transfer reactions associated with H_2_O_2_ reduction or oxidation. These processes generate a concentration-dependent electrochemical response that enables quantitative H_2_O_2_ determination. The incorporation of reduced graphene oxide (RGO) provides an efficient conductive network that facilitates electron transport between the electroactive oxide sites and the electrode surface, thereby enhancing the electrochemical response and analytical sensitivity. Accordingly, MnO_2_–Co_3_O_4_@RGO nanocomposites represent promising electrode materials for non-enzymatic H_2_O_2_ sensing in food products, edible oils, and other complex matrices relevant to oxidative processes. The novelty of the present work lies in the rational integration of MnO_2_, Co_3_O_4_, and RGO into a conductive composite sensing interface, combining the redox-active and electrocatalytic properties of the metal oxides with the efficient electron-transport characteristics of RGO. The resulting composite electrode was systematically evaluated for non-enzymatic H_2_O_2_ detection and compared with the corresponding individual components and related composite configurations to elucidate their respective contributions to the overall sensing performance.

## 2. Experiments

### 2.1. Materials, instruments and methods

Multi-walled carbon nanotubes (MWCNTs) and graphite were purchased from Sigma-Aldrich, and paraffin oil was obtained from PGi Chemicals. Potassium hexacyanoferrate was purchased from Fisher Chemical, potassium ferricyanide from LOBA Chemie PVT. Ltd, and potassium chloride from Chem Lab. Potassium permanganate (KMnO_4_) was obtained from ZA-chem, cobalt nitrate (Co(NO_3_)_2_·6H_2_O) from Piochem, and hydrogen peroxide from ZA-chem. Ascorbic acid, ammonium chloride, d-Glucose, copper sulphate pentahydrate, sodium chloride, urea, and copper sulphate were purchased from Piochem. Lead nitrate was obtained from Power Chemical, and phosphate buffer tablets were purchased from LOBA Chemie. All chemicals were used as received without further purification.

High-resolution transmission electron microscopy (HRTEM, FEI Tecnai T20) was employed to obtain 2D nanoscale images of the nanocomposite structure. Additionally, the functional groups present on the modified electrode surfaces were analyzed using Fourier-transform infrared (FTIR) spectroscopy over a spectral range of 400–4000 cm^−1^ using a Thermo Scientific Nicolet iS10. X-ray diffraction (XRD) analysis using Cu Kα radiation (*λ* = 1.5418 Å) over a 2*θ* scanning range of 4–80° was performed to investigate the crystallographic structure and average crystallite size of the synthesized materials.

Scanning electron microscopy (SEM) analysis was performed using a field-emission scanning electron microscope (Quanta FEG 250) operated at an accelerating voltage of 5.0–10 kV, and EDAX analysis was performed using METEK EDAX. TEM analysis (JEOL JEM-2100, 200 kV) was used to examine the internal structure and nanoscopic dispersion of the nanocomposite components.

Electrochemical measurements were performed using a Corrtest Potentiostat/Galvanostat, CS350H, and a Palmsens-4 potentiostat electrochemical workstation operated using PSTrace version 5.11. A digital pH meter (ADWA Instruments, Hungary) was used for all pH measurements.

### 2.2. Reduced graphene oxide (RGO), MnO_2_, and Co_3_O_4_ nanomaterials synthesis

A modified Hummers' method was used for the synthesis of graphene oxide (GO).^36^ Briefly, 3.0 g of graphite flakes were dispersed in a concentrated H_2_SO_4_/H_3_PO_4_ acid mixture (160 : 40 mL). Subsequently, 15 g of KMnO_4_ was gradually added under ice-bath conditions (5.0 °C) to control the reaction rate. The mixture was then heated to 60 °C and continuously stirred for 24 h to facilitate oxidation. After cooling to room temperature, 5.0 mL of 30% H_2_O_2_ was added to quench the oxidation reaction, resulting in a noticeable color change. The resulting suspension was kept overnight under ambient conditions and subsequently washed repeatedly with distilled water by centrifugation until a neutral pH was achieved. The obtained GO was then dried at room temperature.

RGO was prepared by thermal reduction of GO. The GO was rapidly heated to 300 °C for approximately 30 s, resulting in the removal of oxygen-containing functional groups and concomitant exfoliation and expansion of the material.

Cobalt oxide (Co_3_O_4_) nanoparticles were synthesized using a hydrothermal method. Co(NO_3_)_2_·6H_2_O was dissolved in 50 mL of double-distilled water. Subsequently, 20 mL of ammonia solution was slowly introduced under continuous stirring, and the mixture was maintained for 12 h. The resulting solution was transferred into a 200 mL Teflon-lined stainless-steel autoclave and heated at 200 °C for 10 h. After cooling, the obtained product was calcined at 600 °C for 7.0 h to obtain crystalline Co_3_O_4_ nanoparticles, followed by washing with ethanol and drying.

Manganese dioxide (MnO_2_) nanoparticles were synthesized through a redox reaction in aqueous solution. Initially, 0.0158 g of KMnO_4_ was dissolved in 75 mL of double-distilled water and stirred for 2 h. Separately, 0.0931 g of sodium thiosulfate was dissolved in 25 mL of distilled water. The sodium thiosulfate solution was then added dropwise to the KMnO_4_ solution at room temperature. The reaction was accompanied by a rapid color transition from purple to yellow-brown and finally to dark brown, indicating the progress of the redox reaction. The resulting suspension was collected and processed for further use.

### 2.3. Preparation of carbon paste electrode (CPE) modified with nanomaterials

The carbon paste electrode (CPE) was prepared by thoroughly mixing 750 mg of graphite powder with 250 µL of paraffin oil as the binder. The components were manually homogenized until a uniform, smooth, and mechanically stable carbon paste was obtained. The resulting paste was carefully packed into the cavity of the electrode body and compacted to ensure good electrical contact and a reproducible electrode surface. The working electrode had a circular diameter of 5.0 mm, corresponding to a geometric surface area of approximately 0.196 cm^2^. Aqueous dispersions of the RGO, MnO_2_, Co_3_O_4_, and the MWCNTs were individually prepared at a concentration of 5.0 mg mL^−1^ in distilled water. Each suspension was ultrasonicated for 20 min to ensure homogeneous dispersion.

For the fabrication of nanocomposites, equal volumes of the individual suspensions were mixed in a 1 : 1 : 1 ratio to obtain MnO_2_–Co_3_O_4_@RGO and MnO_2_–Co_3_O_4_@MWCNTs composites. The mixtures were further sonicated for an additional 20 min to promote uniform distribution and strong interaction among the components.

A defined volume (30 µL) of the prepared nanocomposite suspension was then drop-cast onto the surface of carbon paste electrode (CPE) and allowed to dry under ambient conditions, forming a uniform modified layer. The resulting electrodes were used as the working electrodes for subsequent electrochemical measurements.

### 2.4. Electrochemical characterization and sensing measurements

For electrochemical characterization, electrochemical impedance spectroscopy (EIS) and cyclic voltammetry (CV) measurements were conducted using a conventional three-electrode configuration comprising a modified carbon paste electrode as the working electrode, an Ag/AgCl reference electrode (3.0 M KCl), and a Pt-wire as the counter electrode. The interfacial charge-transfer properties of the electrode surfaces were evaluated using 0.1 M KCl containing 5.0 mM [Fe(CN)_6_]^3−/4−^ as the standard redox probe solution. EIS measurements were performed over a frequency range of 0.1 Hz to 10 kHz using an AC perturbation amplitude of 5.0 mV. CV measurements were conducted at a scan rate of 50 mV s^−1^ over a potential range of −0.4 to +1.0 V. The resulting Nyquist plots were analyzed to evaluate the charge-transfer resistance (*R*_ct_) and assess changes in interfacial electron-transfer kinetics following electrode modification.

CV and chronoamperometric (CA) measurements were subsequently performed to evaluate the electrochemical response of the fabricated electrodes toward H_2_O_2_. CV measurements were conducted at a scan rate of 50 mV s^−1^ over a potential range of −0.6 to +1.2 V in phosphate buffer solution (PBS, pH 7.4) to investigate the electrochemical behavior and electrocatalytic response toward H_2_O_2_. Chronoamperometric measurements were performed under continuous stirring by applying a constant potential of +0.9 V. The current response was recorded at different H_2_O_2_ concentrations to construct the calibration relationship and evaluate the analytical sensitivity and response characteristics of the developed sensor.

### 2.5. Real sample preparation and analysis

Food samples, including burger, salami, and cheese, were prepared for H_2_O_2_ analysis using a standard sample preparation procedure. Briefly, 5.0 g of each sample was finely ground and homogenized, and a known concentration of H_2_O_2_ was spiked into each sample prior to extraction. Subsequently, 50 mL of phosphate buffer solution (PBS) was added, and the samples were subjected to continuous magnetic stirring and sonication at 75 °C for 25 min to facilitate sample homogenization and transfer of H_2_O_2_ into the aqueous phase. The resulting suspension was then filtered to remove solid residues, and the filtrate was collected for subsequent electrochemical analysis.

For analysis, 500 µL of the filtrate was diluted with 4500 µL of phosphate buffer solution and analyzed using chronoamperometry. The standard addition method was employed to evaluate the accuracy and reliability of the proposed sensor in the complex food matrices. The analytical performance was assessed based on the recovery of the spiked H_2_O_2_.

For oil sample analysis, commercially purchased palm oil was heated at 150 °C for 2.0 h, and this heating procedure was repeated three times. Subsequently, 1.0 g of the heated oil sample was spiked with a known concentration of H_2_O_2_ and mixed with 5.0 mL of isopropanol. The mixture was shaken thoroughly to obtain a homogeneous solution. Then, 10 µL of the prepared oil sample was added to 5000 µL of phosphate buffer solution and analyzed electrochemically for H_2_O_2_. The accuracy of the method was evaluated based on the recovery of the spiked H_2_O_2_.

## 3. Results and discussion

### 3.1. Material characterizations and feature identifications

First, the Fourier-transform infrared (FTIR) spectroscopy was used to investigate the structural features and functional groups of the synthesized nanomaterials and their composites (Fig. 1A). The spectra were recorded for each individual nanomaterials as well as for the MnO_2_–Co_3_O_4_@RGO and MnO_2_–Co_3_O_4_@MWCNTs nanocomposites. As a result, the FTIR spectrum of MnO_2_ exhibited characteristic absorption bands in the 500–750 cm^−1^ region, which can be assigned to the Mn–O stretching vibrations. For Co_3_O_4_, characteristic bands at approximately 560–590 cm^−1^ were associated with Co–O stretching vibrations. In contrast, the MWCNTs spectrum exhibited bands associated with oxygen-containing surface functionalities, including a broad O–H stretching band at approximately 3400 cm^−1^, a band around 1630 cm^−1^ associated with carbonyl/carbon-based surface vibrations, and C–O stretching vibrations in the 1050–1250 cm^−1^ region.

**Fig. 1:**
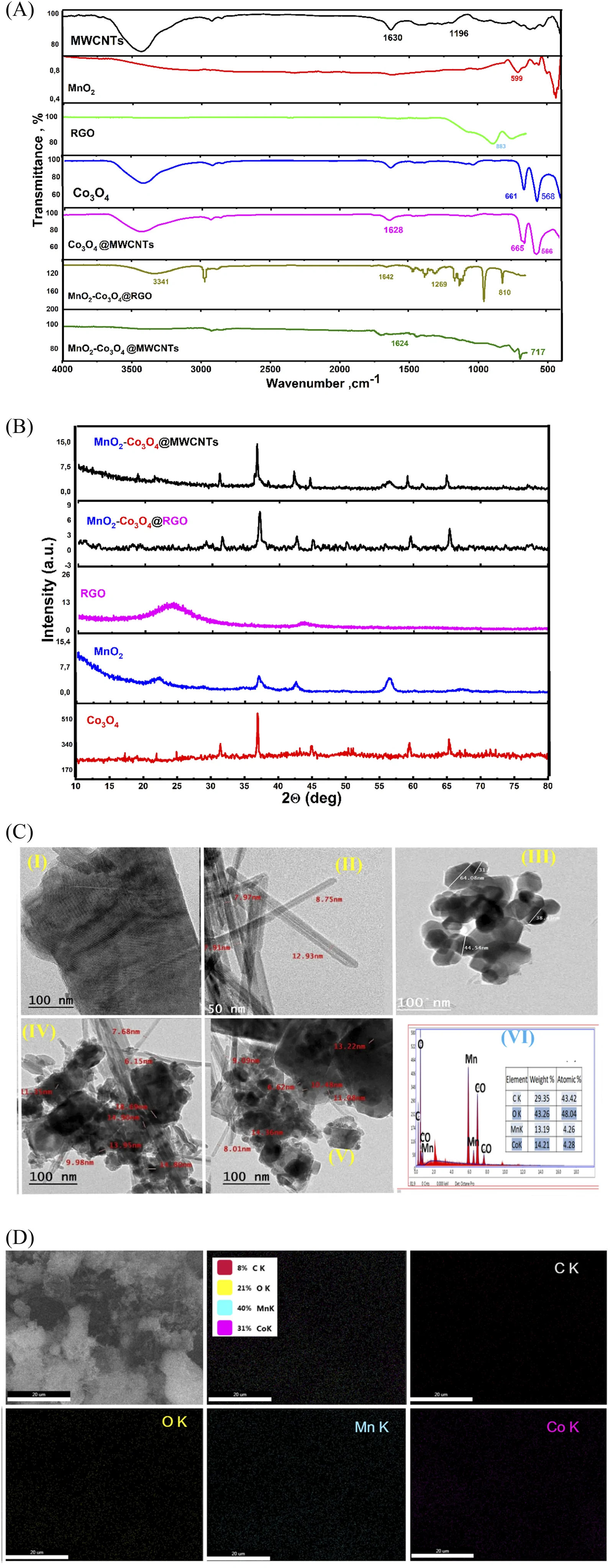
(A) FTIR spectra of the MnO_2_, Co_3_O_4_, RGO, MWCNT, MnO_2_–Co_3_O_4_@RGO and MnO_2_–Co_3_O_4_@MWCNTs. (B) X-ray diffraction (XRD) patterns of prepared individual nanomaterials and nanocomposite. (C) TEM images of (I) RGO, (II) MnO_2_, (III) Co_3_O_4_, (IV) MnO_2_–Co_3_O_4_, and (V) MnO_2_–Co_3_O_4_@RGO; and (VI) EDX spectrum of the MnO_2_–Co_3_O_4_@RGO nanocomposite. (D) Elemental mapping obtained from the EDS analysis of the selected area in the SEM image of the MnO_2_–Co_3_O_4_@RGO nanocomposite.

The FTIR spectrum of the MnO_2_–Co_3_O_4_@MWCNTs nanocomposite exhibited a broadened band in the 500–700 cm^−1^ region, which can be attributed to the overlapping Mn–O and Co–O vibrations. In addition, the C=C-related band around 1600–1650 cm^−1^ was retained, supporting the presence of the carbon nanotube component within the composite. Changes in peak intensity and partial overlap of the characteristic bands of MnO_2_ and Co_3_O_4_ may reflect interactions between the metal oxide components and the carbon framework, consistent with the formation of the composite structure.

For the MnO_2_–Co_3_O_4_@RGO composite, the FTIR spectrum exhibited a combined metal–oxygen band in the 500–700 cm^−1^ region, together with characteristic RGO-related features, including the C=C band at approximately 1600–1650 cm^−1^ and a broad O–H band in the 3300–3500 cm^−1^ region. The presence of these characteristic bands, together with changes in their relative intensities and positions, supports the incorporation of MnO_2_ and Co_3_O_4_ within the RGO-based composite. The observed spectral changes are consistent with interfacial interactions between the metal oxides and the carbonaceous support.

Second, the crystalline and structural characterization of the synthesized nanomaterials was studied by the powder X-ray diffraction (XRD) analysis (Fig. 1B). The diffraction patterns exhibited the characteristic reflections of RGO, MnO_2_, and Co_3_O_4_. The XRD pattern of RGO showed a broad diffraction feature centered at 2*θ* ≈ 24–26°, corresponding to the (002)-related reflection of graphitic carbon. The broad nature of this feature is consistent with the partially disordered and few-layer character of the RGO structure.

The XRD pattern of MnO_2_ exhibited characteristic diffraction peaks at 2*θ* values of 21.9°, 36.99°, 42.79°, 56.44°, and 67.16°, which can be indexed to the (110), (211), (301), (600), and (002) planes, respectively. These reflections are consistent with the tetragonal α-MnO_2_ phase (JCPDS no. 44-0141), supporting the formation of crystalline MnO_2_. For Co_3_O_4_, well-defined diffraction peaks were observed at 2*θ* values of 21.07°, 31.20°, 37.05°, 38.41°, 45.03°, 51.03°, 60.23°, and 66.10°, which can be indexed to the (111), (220), (311), (222), (400), (422), (511), and (440) planes, respectively. These reflections are in good agreement with the standard diffraction data for cubic spinel Co_3_O_4_ (ICSD card no. 98-006-3164), confirming the crystalline spinel structure of the synthesized Co_3_O_4_.^37^

The XRD patterns of the MnO_2_–Co_3_O_4_@MWCNTs and MnO_2_–Co_3_O_4_@RGO nanocomposites retained the characteristic reflections associated with the metal oxide phases together with the corresponding carbon-based components. No additional prominent diffraction peaks attributable to crystalline impurity phases were observed. In the MnO_2_–Co_3_O_4_@RGO composite, the broad carbon-related diffraction feature partially overlapped with the metal oxide reflections. Slight changes in peak broadening and position were observed after composite formation, which may be associated with changes in crystallite characteristics and the local structural environment resulting from interactions between MnO_2_, Co_3_O_4_, and the conductive carbon matrices. Collectively, the XRD results support the successful formation of the MnO_2_–Co_3_O_4_-based nanocomposites.

Third, the TEM 2D-imaging technique was used to investigate the morphology, particle size, and structural features of MnO_2_ nanorods, Co_3_O_4_ nanoparticles, RGO nanosheets, and their corresponding nanocomposites (Fig. 1C). The observed nanoscale morphologies and dispersion characteristics provide nanostructured architectures that will increase the accessible surface area and facilitate interfacial electrochemical processes. The RGO exhibited thin, wrinkled nanosheet-like structures (Fig. 1C(I)), whereas MnO_2_ exhibited a nanorod morphology (Fig. 1C(II)), with a reported size range of approximately 7.9–12.9 nm. These nanoscale structures provide a high accessible surface area and may facilitate exposure of electroactive sites. The Co_3_O_4_ exhibited nanoparticles with a size range of 19.4–34.2 nm (Fig. 1C(III)), together with a relatively well-defined morphology and moderate aggregation.

For the MnO_2_–Co_3_O_4_ composite, the TEM images showed MnO_2_ nanorods and Co_3_O_4_ nanoparticles distributed throughout the composite structure (Fig. 1C(IV)). In the MnO_2_–Co_3_O_4_@RGO nanocomposite, the MnO_2_ nanorods and Co_3_O_4_ nanoparticles appeared to be distributed over the RGO nanosheets (Fig. 1C(V)). The relatively homogeneous distribution of the metal oxide components over the RGO framework may reduce extensive particle aggregation and increase the accessibility of electroactive sites. Thus, the TEM results support the successful integration of the nanoscale metal oxides with the RGO support and reveal a composite architecture favorable for interfacial electrochemical processes.

The morphology and elemental composition of the synthesized MnO_2_–Co_3_O_4_@RGO nanocomposite were further investigated using scanning electron microscopy (SEM), energy-dispersive X-ray spectroscopy (EDX) as shown in Fig. 1C(VI), and elemental mapping (shown in Fig. 1D). Thus, the SEM demonstrated the characteristic morphology of the nanocomposite, with the MnO_2_ and Co_3_O_4_ components distributed over the wrinkled RGO sheets. The RGO framework provides a conductive carbonaceous support that can facilitate the dispersion of the metal oxide components within the composite structure.

The EDX spectrum provided the elemental composition of the nanocomposite, whereas Mn, and Co, C, O, supporting the incorporation of MnO_2_ and Co_3_O_4_ within the RGO-based material. Furthermore, the elemental mapping images demonstrated the spatial distribution of Mn, Co, C, and O throughout the selected region. The relatively uniform distribution of the Mn and Co signals over the carbonaceous framework provides further evidence of the homogeneous integration of the metal oxide components within the MnO_2_–Co_3_O_4_@RGO nanocomposite.

### 3.2. Electrochemical characterization of the synthesized nanomaterials

The electrochemical properties of the modified electrode surfaces were systematically evaluated using the CV and EIS, whereas a standard redox probe solution containing 5.0 mM [Fe(CN)_6_]^3−/4−^in 0.1 M KCl was used to evaluate the electrochemical performance of unmodified alongside different modified electrodes. Given the favourable electrochemical properties of metal oxide (MO) nanostructures, a series of metal oxides (ZnO, NiO, MnO_2_, and Co_3_O_4_) was initially tested, as shown in Fig. 2A and B. The unmodified CPE provided relatively low anodic and cathodic peak currents, together with a high charge-transfer resistance (*R*_ct_), indicating relatively sluggish electron-transfer kinetics at the electrode/electrolyte interface. Modification with the individual metal oxides resulted in increased redox currents and decreased *R*_ct_ values. Among the investigated individual metal oxides, MnO_2_ and Co_3_O_4_ produced particularly pronounced improvements in the electrochemical response, as reflected by their higher redox currents and lower *R*_ct_ values compared with the bare CPE (see Table 1). These results indicate that incorporation of the metal oxide nanostructures substantially improves the interfacial electron-transfer characteristics of the CPE.

**Fig. 2:**
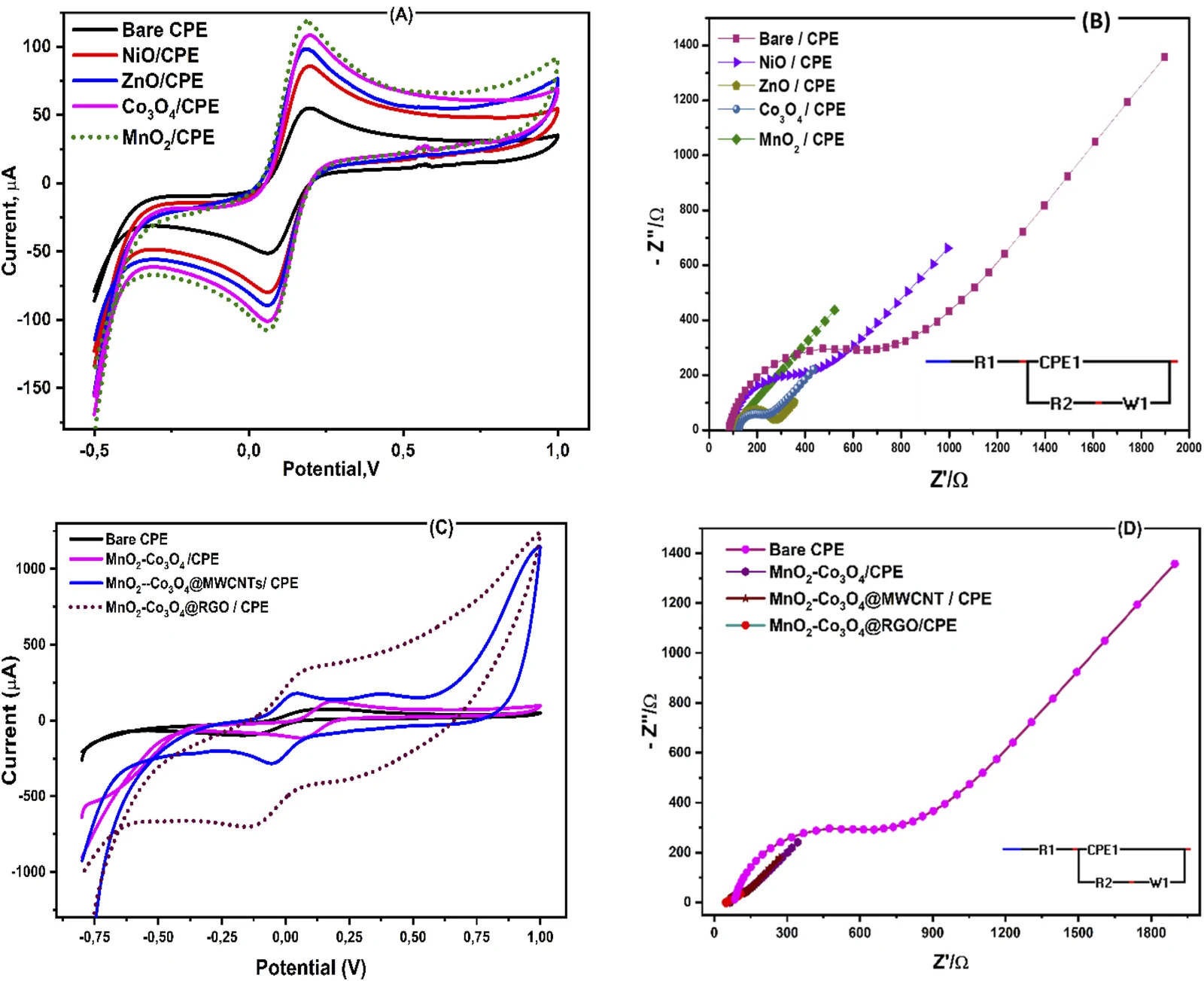
(A) CVs and (B) Nyquist plots of the bare and modified carbon paste electrodes containing individual metal oxides (ZnO, NiO, MnO_2_, and Co_3_O_4_). (C) CVs and (D) Nyquist plots of the bare, MnO_2_–Co_3_O_4_, MnO_2_–Co_3_O_4_@MWCNTs, and MnO_2_–Co_3_O_4_@RGO-modified carbon paste electrodes. The inset shows the equivalent circuit used for fitting the EIS spectra, where *R*_s_ represents the solution resistance, *R*_ct_ the charge-transfer resistance, CPE the constant-phase element, and *W* the diffusion-related Warburg element. All experiments were performed at a scan rate of 50 mV s^−1^ using 5.0 mM [Fe(CN)_6_]^4−/3−^ in 0.1 M KCl as the supporting electrolyte.

**Table 1:** Summary of (A) CVs and (B) EISs (Nyquist plots) parameters for bare and modified CPEs with the synthesized nanomaterials

Electrode type	*I*_a_	*I*_c_	*E*_oxd._	*E*_red._	*R*_s_	*R*_ct (1)_	CPE	*n*	W
	(µA)	(µA)	(V)	(V)	(Ω)	(Ω)	µF		(Ω)
Unmodified CPE	60.0	−48.2	0.189	0.067	92	900	0.127	0.96	324
ZnO/CPE	96.0	−79.0	0.195	0.058	102	700	0.132	0.89	332
NiO/CPE	90.2	−71.0	0.195	0.065	122	180	0.36	0.88	341
Co_3_O_4_/CPE	111.8	−103.0	0.195	0.056	125	150	0.39	0.82	352
MnO_2_/CPE	117.9	−94.0	0.195	0.065	115	100	0.42	0.92	363
MnO_2_–Co_3_O_4_/CPE	145.0	−124.0	0.179	0.081	65	70	0.46	0.89	378
MnO_2_–Co_3_O_4_@MWCNT/CPE	205.0	−289.0	0.044	−0.05	65	60	0.51	0.93	382
MnO_2_–Co_3_O_4_@RGO/CPE	360.0	−698.0	0.090	−0.13	47	35	0.63	0.89	399

Further enhancement was observed for the MnO_2_–Co_3_O_4_ composite (Fig. 2C and D). Compared with the individual metal oxide-modified electrodes, the composite exhibited higher redox currents and a lower *R*_ct_ (Table 1), indicating more favorable interfacial charge-transfer behavior. This improvement may be associated with the complementary electrochemical characteristics of MnO_2_ and Co_3_O_4_ and their combined contribution to the modified electrode interface.

The incorporation of conductive carbon nanomaterials (MWCNTs and RGO) resulted in a further enhancement of the electrochemical response. The MnO_2_–Co_3_O_4_@MWCNTs/CPE exhibited substantially higher redox currents and a lower *R*_ct_ than the MnO_2_–Co_3_O_4_/CPE. This behavior is consistent with improved electron transport through the conductive MWCNTs network and enhanced electrical communication between the metal oxide components and the electrode surface.

Among all tested electrode configurations, the MnO_2_–Co_3_O_4_@RGO/CPE exhibited the highest redox-current response and the lowest *R*_ct_ (Table 1). The substantial decrease in *R*_ct_ relative to the bare CPE indicates a pronounced improvement in interfacial charge-transfer characteristics. This behavior can be attributed to the combined contribution of the electroactive MnO_2_ and Co_3_O_4_ nanostructures and the conductive RGO framework, which provides an effective interfacial pathway for electron transport. The corresponding changes in the oxidation and reduction potentials further indicate modification of the electrode/electrolyte interfacial electrochemical behavior following incorporation of the RGO component.

The EIS data were further analyzed by fitting the Nyquist plots using the corresponding equivalent circuit. The fitted constant phase element (CPE) parameters varied among the different electrode configurations, reflecting changes in the interfacial capacitive behavior and surface characteristics following electrode modification. Similarly, the variation in the Warburg parameter (*W*) indicates changes in the diffusion-related contribution to the overall interfacial impedance. Therefore, the combined CV and EIS results demonstrate that incorporation of MnO_2_ and Co_3_O_4_, particularly in combination with conductive RGO, substantially modifies the interfacial electrochemical properties of the CPE. The MnO_2_–Co_3_O_4_@RGO/CPE exhibited the highest measured redox response and the lowest charge-transfer resistance among the investigated electrodes, indicating favorable interfacial electron-transfer characteristics. These properties support the selection of the MnO_2_–Co_3_O_4_@RGO-modified electrode for subsequent investigation of its non-enzymatic electrochemical response toward H_2_O_2_.

### 3.3. Scan rate effect and electroactive surface area calculation

Electrochemical properties of the different modified electrodes were systematically investigated using cyclic voltammetry with a wide range of scan rates (as shown in Fig. 3(A–F)). Both of cathodic and anodic peak currents increased progressively with increasing the scan rate (see Fig. 4(A–F)), indicating an enhancement in the electron transfer process. Furthermore, a slight shift in the peaks potential of reduction and oxidation toward more negative and positive values, respectively, was observed at higher scan rates, which can be owing to increased polarization and kinetic limitations of the redox process. Subsequently, as shown in Fig. 5, a linear relationship was obtained between the square root of the scan rate (*ν*^1/2^) and the peak current (*I*_p_), suggesting that the electrochemical redox reactions of the redox probe is driven by diffusion forces (diffusion-controlled process). The improved current response at higher scan rates approves the fast electron transfer kinetics and efficient charge transport properties of the modified electrodes. In particular, the nanocomposite-based electrodes (MnO_2_–Co_3_O_4_@MWCNTs and MnO_2_–Co_3_O_4_@RGO) exhibited a more pronounced increase in peak currents when its generated values were compared with the bare electrode, highlighting the role of conductive carbon matrices in facilitating rapid electron mobility. To estimate the electroactive surface area, the Randles–Ševčík equation was applied:*I*_p_ = (2.69 × 10^5^) *n*^3/2^*CAD*^1/2^*v*^1/2^where *I*_p_ represents the oxidation peak current, *C* is the concentration of the electroactive species (mol cm^−3^), *A* is the electroactive surface area (cm^2^) *n* is the number of electrons involved in the redox reaction, *D* is the diffusion coefficient (cm^2^ s^−1^), and *ν* is the scan rate (V s^−1^).

**Fig. 3:**
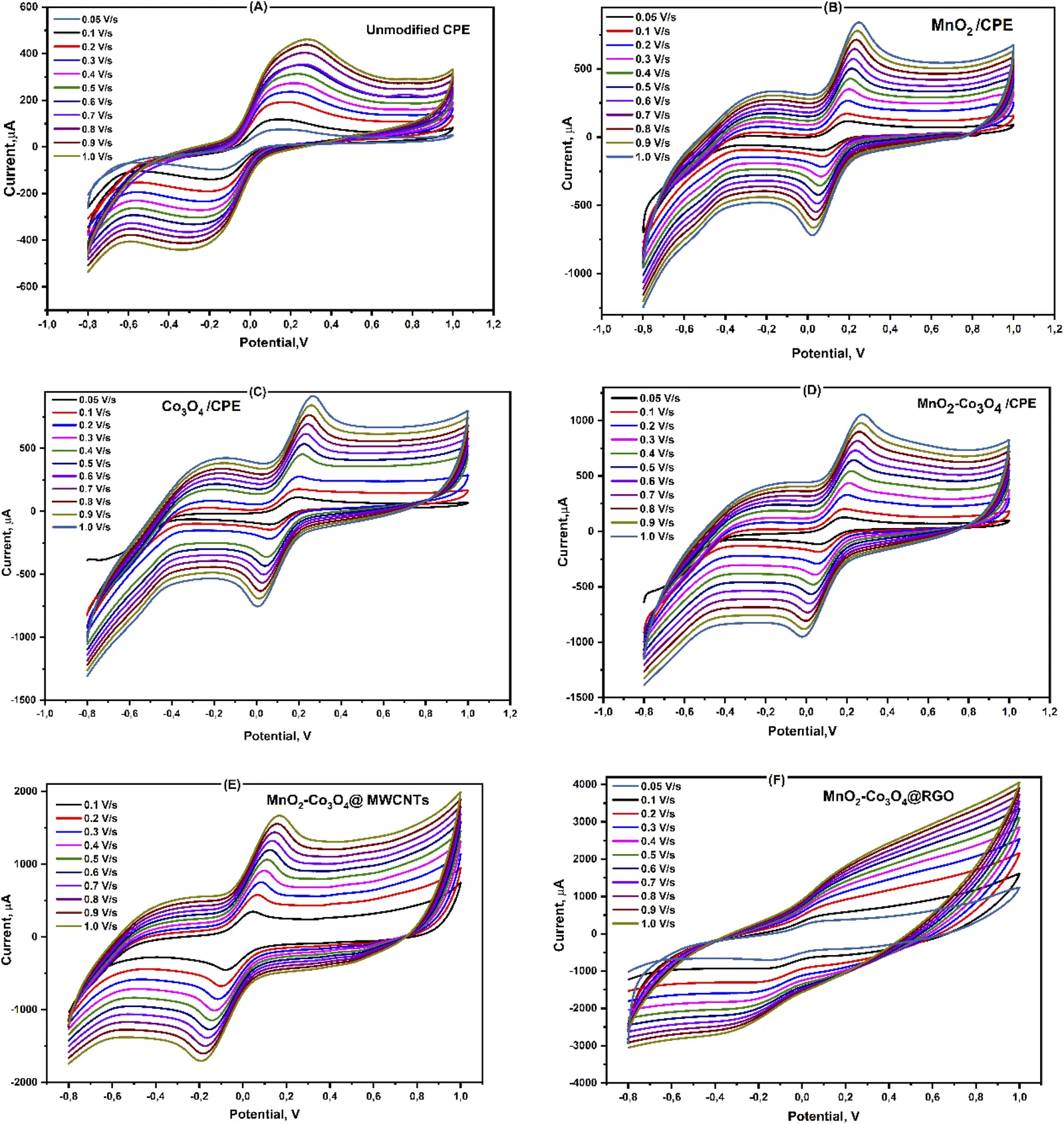
Cyclic voltammograms curves at different scan rate for (A) bare (unmodified CPE), (B) MnO_2_, (C) Co_3_O_4_, (D) MnO_2_–Co_3_O_4_, (E) MnO_2_–Co_3_O_4_@MWCNTs and (F) MnO_2_–Co_3_O_4_@RGO modified CPEs.

**Fig. 4:**
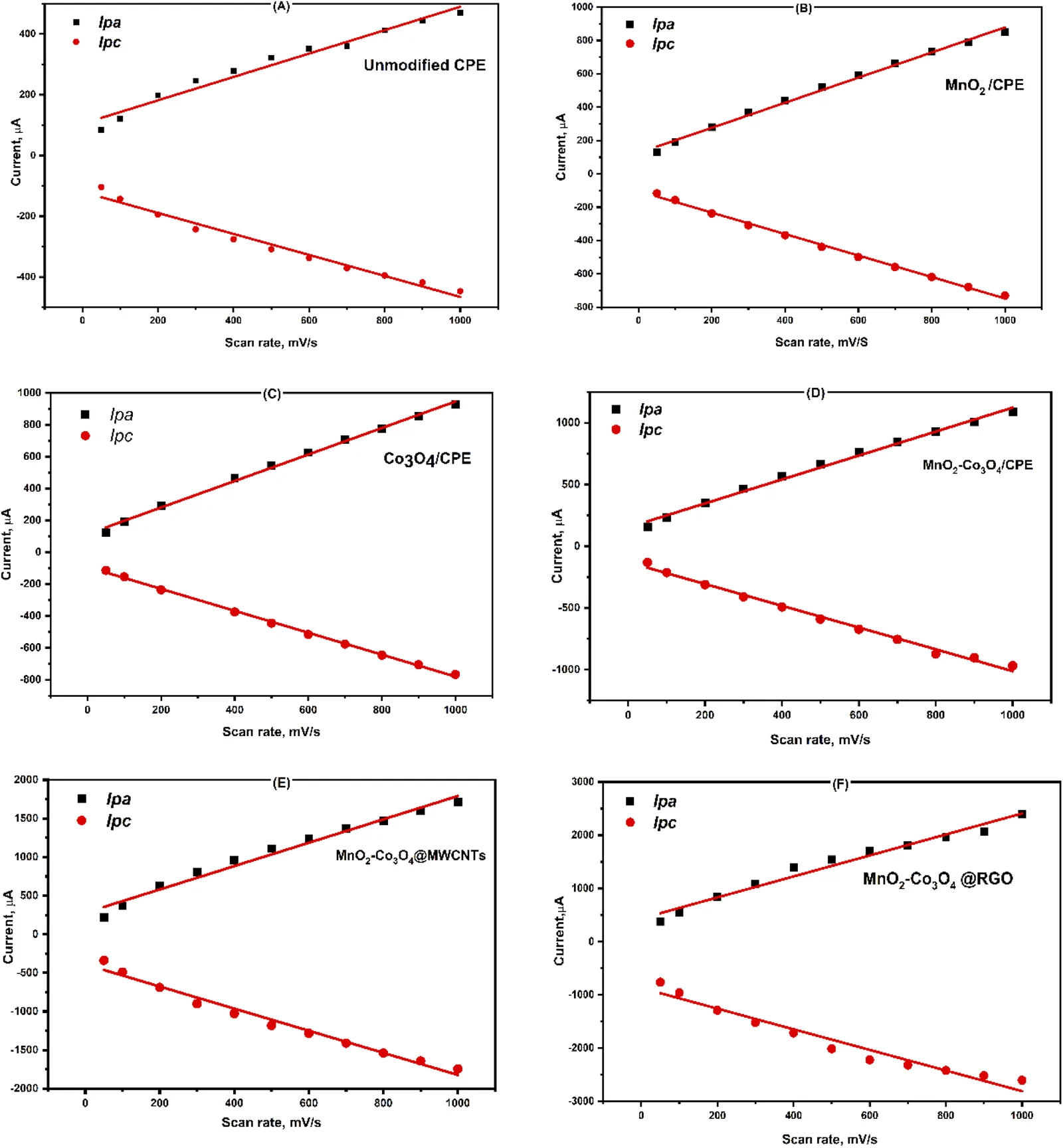
Linear relationship between the scan rate increase and the redox peak currents anodic and cathodic (*I*_pa_, and *I*_pc_, respectively) for (A) the unmodified electrode *vs.* (B) MnO_2_, (C) Co_3_O_4_, (D) MnO_2_–Co_3_O_4_, (E) MnO_2_–Co_3_O_4_@MWCNTs and (F) MnO_2_–Co_3_O_4_@RGO modified CPEs.

**Fig. 5:**
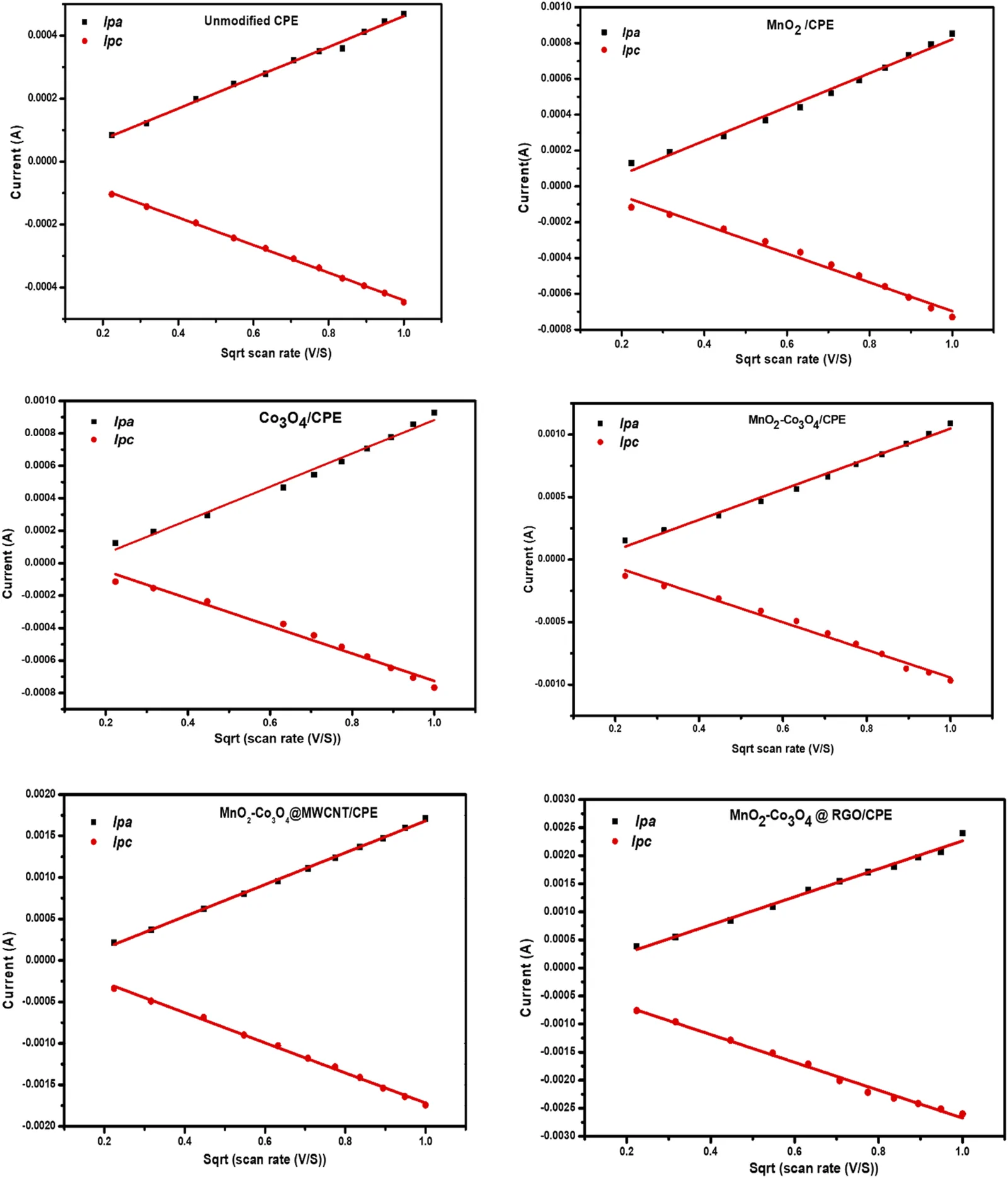
The linearity calibration plot of the square root of scan rate (*ν*^1/2^) *vs.* the peak current.

The bare CPE exhibited a relatively low active surface area of 0.137 cm^2^, while modification with individual materials led to a noticeable increase, reaching 0.270 cm^2^ for MnO_2_, and 0.295 cm^2^ for Co_3_O_4_-modified electrodes and for MnO_2_–Co_3_O_4_ mixture enhanced to be 0.3504. Notably, the MnO_2_–Co_3_O_4_@MWCNT/CPE evaluated 0.5515 cm^2^ and the MnO_2_–Co_3_O_4_@RGO nanocomposite electrode demonstrated a significantly enhanced electroactive surface area of approximately 0.7181 cm^2^, which is nearly five times higher than that of the bare electrode.

This substantial increase in electroactive surface area is attributed to the synergistic integration of conductive RGO sheets with catalytically active MnO_2_ and Co_3_O_4_ nanoparticles, resulting in a highly abundant active sites. The enlarged electroactive surface accelerates the electron transfer at the electrode/electrolyte interface, thereby contributing to the superior electrochemical performance.

### 3.4. Non-enzymatic electrochemical detection of H_2_O_2_

The objective of this study was to develop a sensitive non-enzymatic electrochemical platform for H_2_O_2_ determination using nanostructured electrode materials. Among the investigated electrodes, the MnO_2_–Co_3_O_4_@RGO-modified electrode exhibited the highest electrochemical response and was therefore selected for peroxide analysis. The enhanced response is associated with the electrochemical activity of the MnO_2_ and Co_3_O_4_ phases together with the conductive RGO framework, which can facilitate electron transport and increase the accessibility of electroactive regions at the electrode interface.

Subsequently, the effect of electrolyte pH on the H_2_O_2_ electrochemical response was systematically investigated in phosphate-buffered saline (PBS) over the pH range of 5.0–9.0 using cyclic voltammetry (CV). As shown in Fig. 6, the oxidation current increased progressively with increasing pH and reached a maximum at pH 7.4. Further increasing the pH resulted in a decrease in the current response. This pH dependence indicates that the electrochemical oxidation of H_2_O_2_ at the modified electrode is influenced by the protonation state and interfacial reaction conditions. Therefore, pH 7.4 was selected as the optimum electrolyte condition for subsequent experiments. The use of a near-neutral medium also provides a practically relevant condition for H_2_O_2_ determination in aqueous samples.

**Fig. 6:**
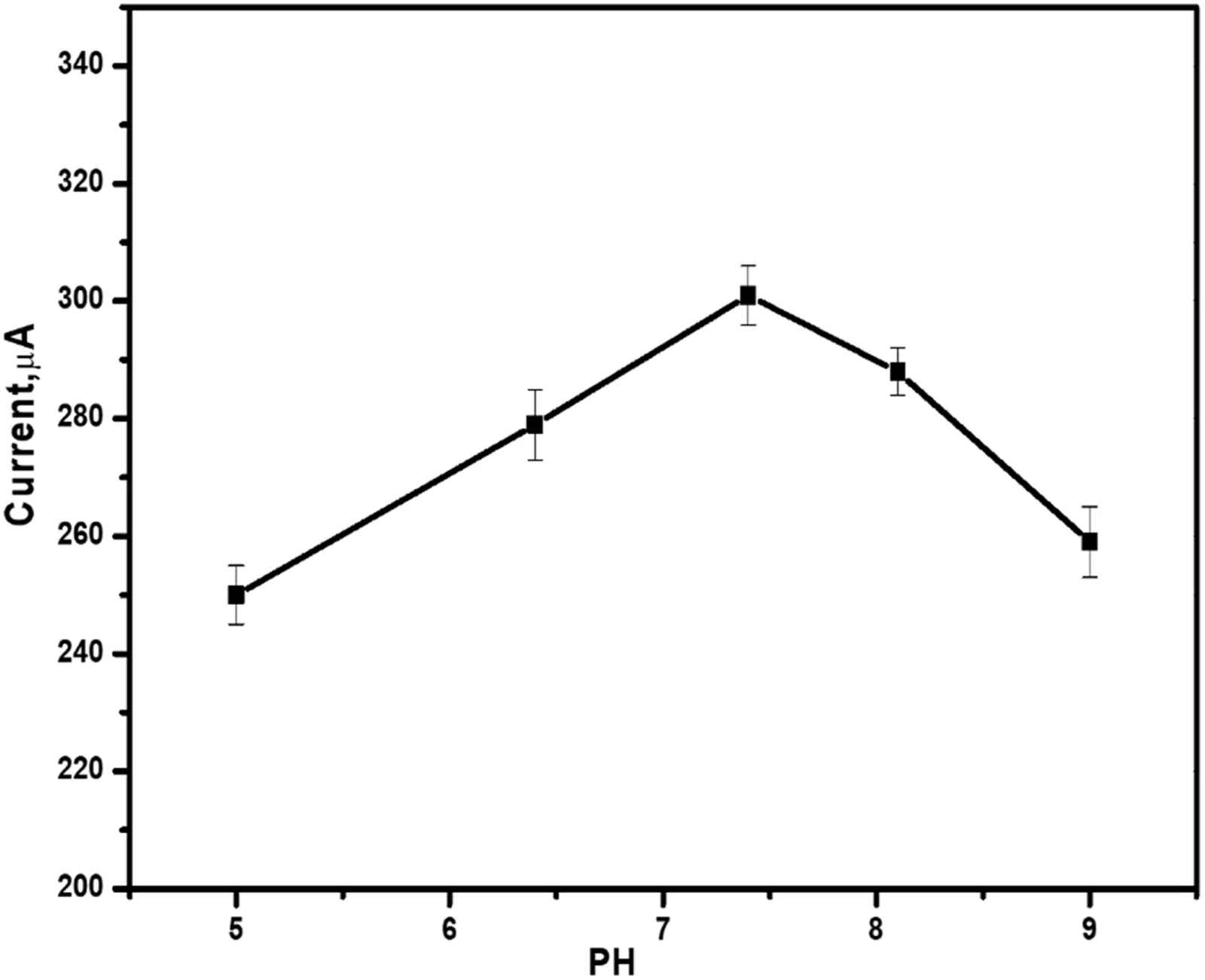
PH Effect study of PBS buffer on the oxidation peaks current response of peroxide using the MnO_2_/Co_3_O_4_@RGO modified electrode.

The effect of H_2_O_2_ concentration was subsequently investigated by adding increasing concentrations of H_2_O_2_ to PBS at pH 7.4. The corresponding cyclic voltammograms were recorded over the potential range from −0.8 to +1.2 V at a scan rate of 50 mV s^−1^ (Fig. 7).

**Fig. 7:**
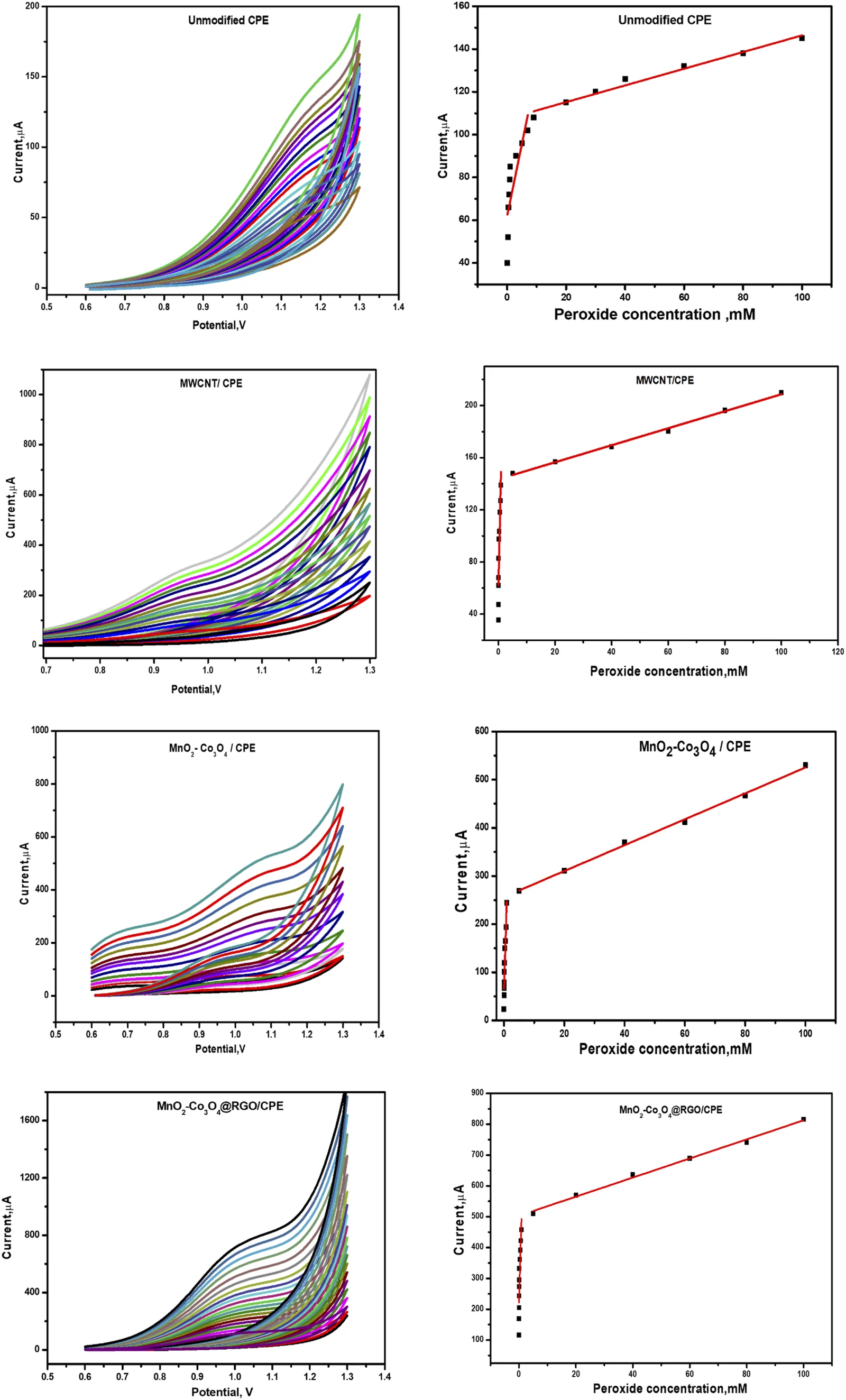
The CVs curves and fitting linear ranges of unmodified, MnO_2_/Co_3_O_4_, and MnO_2_/Co_3_O_4_@RGO modified CPE using solution consist of PBS buffer (pH = 7.4) and different concentrations of peroxide.

Among the tested materials (bare, MWCNT, MnO_2_–Co_3_O_4_, and MnO_2_–Co_3_O_4_@RGO), the MnO_2_/Co_3_O_4_@RGO nanocomposite-modified electrode evaluated the highest electrocatalytic efficiency toward direct oxidation of peroxide with high sensitivity, wide linear range (two linear concentration ranges of 0.01–5 mM and 5.0–100 mM), with an overall correlation coefficient of *R*^2^ = 0.997 and a calculated detection limit of 2.0 µM. The pronounced H_2_O_2_ response can be attributed to the electrochemical activity of the MnO_2_–Co_3_O_4_ phases, while the RGO component provides a conductive network that supports electron transport across the modified electrode interface. These characteristics make the MnO_2_–Co_3_O_4_@RGO composite suitable for non-enzymatic electrochemical H_2_O_2_ detection.

Chronoamperometric measurements were then performed to further evaluate the analytical performance of the MnO_2_–Co_3_O_4_@RGO-modified carbon paste electrode (CPE). Successive additions of H_2_O_2_ were introduced at fixed 50 s intervals under continuous stirring in PBS (pH 7.4).

The effect of the applied potential on the chronoamperometric response was evaluated at 0.50, 0.55, 0.60, 0.65, 0.70, 0.75, 0.80, 0.85, 0.90, and 1.00 V. As shown in Fig. 8, each H_2_O_2_ addition produced a distinct increase in the oxidation current, confirming the concentration-dependent response of the modified electrode.

**Fig. 8:**
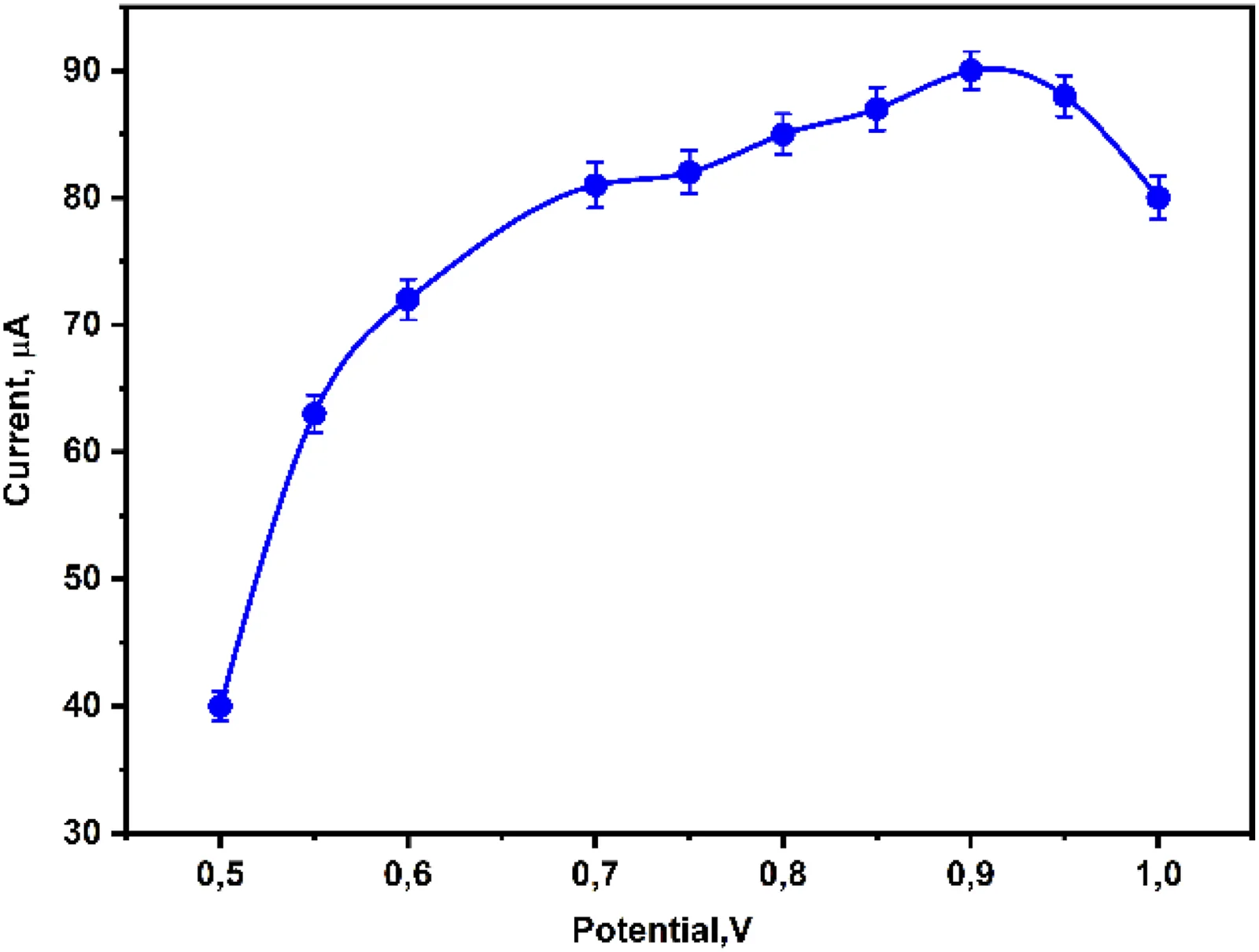
Effect of various applied DC potentials (0.5–1.0 V) on the chronoamperometric response of peroxide oxidation.

The calibration responses obtained at the different applied potentials were compared to determine the potential providing the most favorable analytical response. Among the investigated potentials, 0.90 V produced the highest oxidation current and the most favorable linear correlation between the H_2_O_2_ concentration and the measured current (Fig. 8). Accordingly, 0.90 V was selected as the applied potential for subsequent chronoamperometric measurements.

The chronoamperometric response obtained at 0.90 V and the corresponding calibration curve are presented in Fig. 9A and B, respectively. The MnO–Co_3_O_4_@RGO-modified CPE exhibited a rapid current response following each H_2_O_2_ addition, with a response time of approximately 12 s. A linear response was obtained over the concentration range of 0.1–1800 µM, with a sensitivity of 0.173 µA µM^−1^ and an excellent correlation coefficient (*R*^2^ = 0.996). The limit of detection (LOD) and limit of quantification (LOQ) were calculated according to the ICH approach using the equations: (LOD = 3.3*σ/slope) and (LOQ = 10*σ/slope), respectively where *σ* represents the standard deviation used for the estimation of the analytical noise and *S* is the slope of the calibration curve. Using *σ* = 0.001553 µA and *S* = 0.1726 µA µM^−1^, the calculated LOD and LOQ were 0.03 µM and 0.09 µM, respectively. These values, together with the wide range linear response and rapid signal development, demonstrate the favorable analytical characteristics of the MnO_2_–Co_3_O_4_@RGO-modified CPE for quantitative H_2_O_2_ determination. A comparison of the analytical performance with previously reported electrochemical H_2_O_2_ sensors is presented in Table 2.

**Fig. 9:**
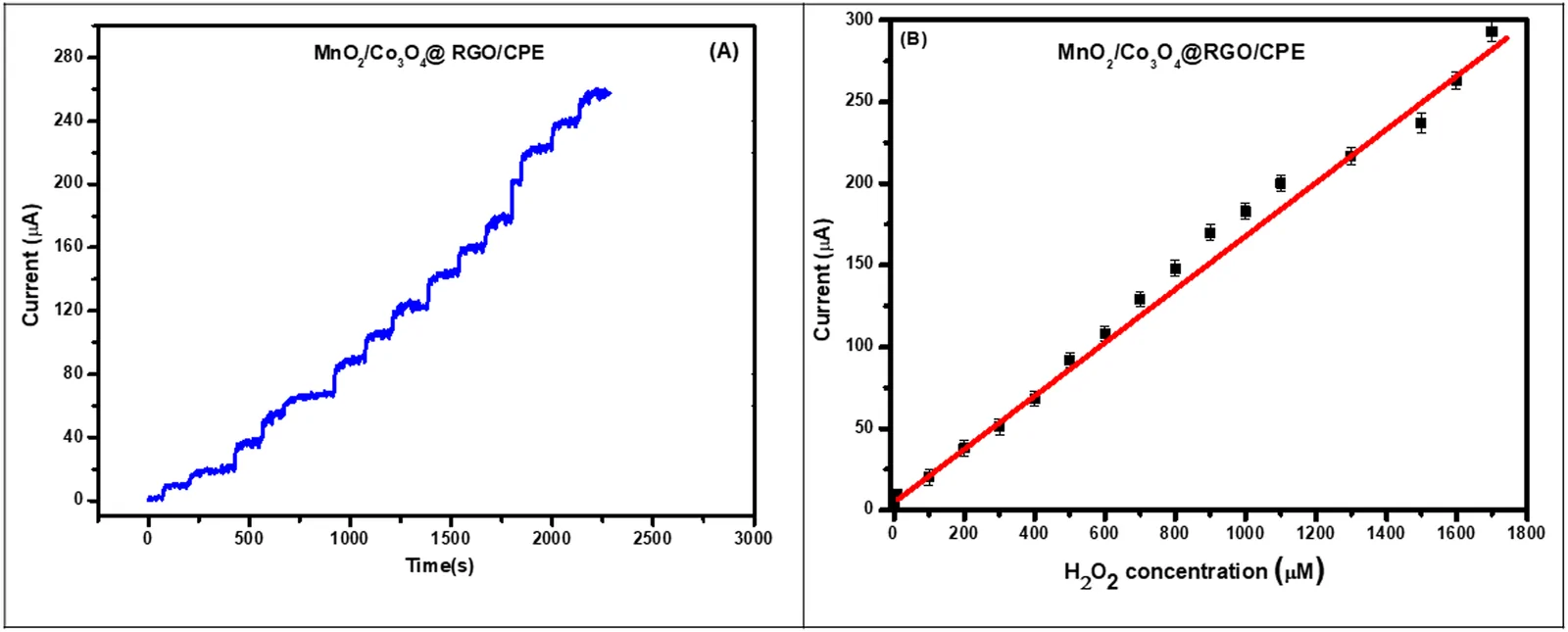
(A) Chronoamperometric (CA) responses (at 0.9 V), and (B) linearity calibration curves of oxidation current *vs.* H_2_O_2_ concentration. The measurements were performed using the MnO_2_–Co_3_O_4_@RGO modified electrodes following the successive addition of different H_2_O_2_ concentrations in PBS buffer (pH 7.4).

**Table 2:** Comparison between MnO_2_/Co_3_O_4_@RGO-modified CPE and previously reported nanomaterials-based working electrodes for peroxide electrochemical determination

Electrode	Sensitivity	LOD	Linear range	Ref.
	(µA mM^−1^ cm^−2^)			
Ni_3_B NPs/f-CNFs/GCE	59.08	0.021 µM	0.08 µM–12.6 mM, 14.2–39.5 mM	38
Ni_5.5_Co_1.5_S_6_/GCE	94.7	0.15 µM	5 µM–15.09 mM	39
Ni_3_N/GA/GCE	101.9	1.8 µM	5 µM–75.13 mM	40
3D flower-like Ni_7_S_6_/GCE	37.7	0.15 µM	5 µM–20.5 mM	41
Ni(OH)_2_/ERGO/GCE	711	4.0 µM	20 µM–9.05 mM	42
PCF Ni NP/GCE	43	1.1 µM	3.3 µM–1.7 mM	43
Ni_*x*_Co_3−*x*_N/NG/GCE	2848.7	0.2 µM	0.2 µM–3.50 mM	44
Ni/NiO@C	32.1	0.9 µM	Up to 80.7	45
NP–PdNi/GCE	208.6	2.1 µM	50 µM–1 mM	46
GDC/NiO GCE	107.2	0.001 µM	0.01–3.9 µM	47
CB/nano-NiO/GC	0.008	1.67 mM	5–20 000 µM	48
NiO/αFe_2_O_3_	146.9	0.05 mM	0.5–3 mM	49
NiO/Graphene	591	0.76 µM	0.25–4.75 mM	50
Ni(OH)_2_/RGO/Cu_2_O@Cu	1706.3	0.2 µM	0.5 µM–7.5 mM	51
NHGH/NiCo_2_O_4_	Not given	0.136 µM	1 µM–0.51 mM	52
MnO_2_–Co_3_O_4_@RGO	172.6 µA mM^−1^	0.03 µM	0.1–1800 µM	This work

### 3.5. Mechanism of H_2_O_2_ detection

The proposed mechanism of non-enzymatic H_2_O_2_ detection at the MnO_2_–Co_3_O_4_@RGO-modified electrode is based on the electrochemical oxidation of H_2_O_2_ at the modified electrode interface and the contribution of the metal-oxide phases and RGO to the overall electrode response (Scheme 1). MnO_2_ and Co_3_O_4_ provide electrochemically active oxide surfaces, while the RGO phase provides a conductive carbon network that facilitates electron transport between these domains and the electrode substrate. The MnO_2_ is predominantly composed of Mn(iv), whereas Co_3_O_4_ is a mixed-valence oxide containing Co(ii) and Co(iii). These metal oxides can participate in interfacial electron-transfer processes associated with H_2_O_2_ oxidation. However, the electrochemical data obtained in this study do not support the assignment of specific Mn- or Co-centered oxidation–state transitions or individual elementary catalytic steps. The RGO component provides an electrically conductive pathway within the composite and facilitates electron transport from the electrochemically active interface toward the electrode. The integration of the metal-oxide phases with RGO therefore provides an electrochemically accessible composite interface for H_2_O_2_ oxidation. Under the applied potential of 0.90 V, H_2_O_2_ oxidation generates a faradaic current at the modified electrode, with the magnitude of the current increasing with increasing H_2_O_2_ concentration. The overall anodic process can be represented, in simplified form, as: H_2_O_2_ → O_2_ + 2H^+^ + 2e^−^

**Scheme 1:**
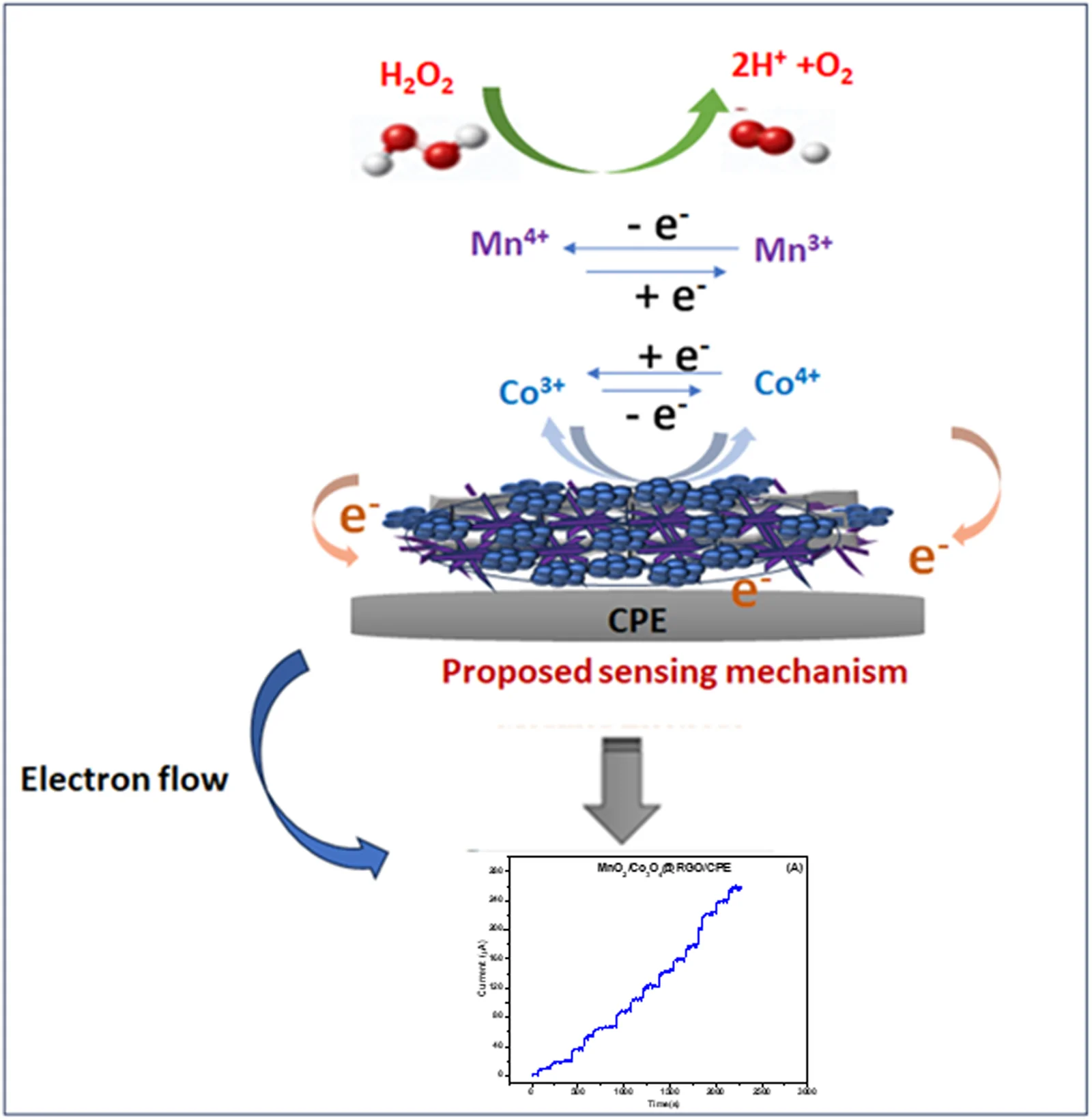
Schematic diagram for peroxide detection mechanism.

The measured chronoamperometric response is therefore attributed to the electrochemical oxidation of H_2_O_2_ at the MnO_2_–Co_3_O_4_@RGO-modified interface, with electron transport through the conductive electrode architecture contributing to the recorded faradaic signal. The concentration-dependent current response, together with the broad linear range, low detection limit, and rapid response observed experimentally, demonstrates the effectiveness of the modified electrode for non-enzymatic H_2_O_2_ determination. Thus, Scheme 1 presents a simplified representation of the sensing process: H_2_O_2_ reaches the electrochemically active MnO_2_–Co_3_O_4_ interface, undergoes anodic electrochemical oxidation under the applied potential, and the generated electrons are transferred through the conductive RGO-containing electrode structure to produce the measurable faradaic current.

### 3.6. Selectivity and interference study

The selectivity of the MnO_2_–Co_3_O_4_@RGO-modified electrode toward the oxidation of H_2_O_2_ was assessed by chronoamperometry in the presence of potentially interfering species. The selected compounds represented constituents or potential contaminants of food and edible-oil matrices, together with electrochemically active species that could potentially affect the measured current under the applied detection conditions. H_2_O_2_ was tested at a concentration of 0.1 mM, while each interferent was introduced at 0.2 mM.

The investigated species included ascorbic acid (AA), citric acid, urea, sucrose, and d-glucose as representative food-related compounds; ammonium chloride (NH_4_Cl), lead nitrate [Pb(NO_3_)_2_], copper(ii) sulfate pentahydrate (CuSO_4_·5H_2_O), sodium chloride (NaCl), and potassium chloride (KCl) as representative inorganic species; and oleic acid (OA) as a representative component of edible-oil matrices. Uric acid (UA) and dopamine (DA) were additionally examined as electroactive compounds to assess the susceptibility of the modified electrode to non-target electrochemical responses. As shown in Fig. 10, the successive addition of the investigated interferents produced no appreciable change in the chronoamperometric current under the applied conditions, whereas the addition of 0.1 mM H_2_O_2_ resulted in a distinct increase in the current response. Importantly, a subsequent addition of H_2_O_2_ produced a clear response comparable to the initial H_2_O_2_ response, indicating that the investigated species did not interfere with H_2_O_2_ detection at the tested concentrations. The absence of pronounced responses to the tested interferents, together with the reproducible response to subsequent H_2_O_2_ addition, demonstrates favorable interference tolerance of the MnO_2_–Co_3_O_4_@RGO-modified electrode. These findings support its potential applicability for H_2_O_2_ determination in complex food- and oil-related matrices.

**Fig. 10:**
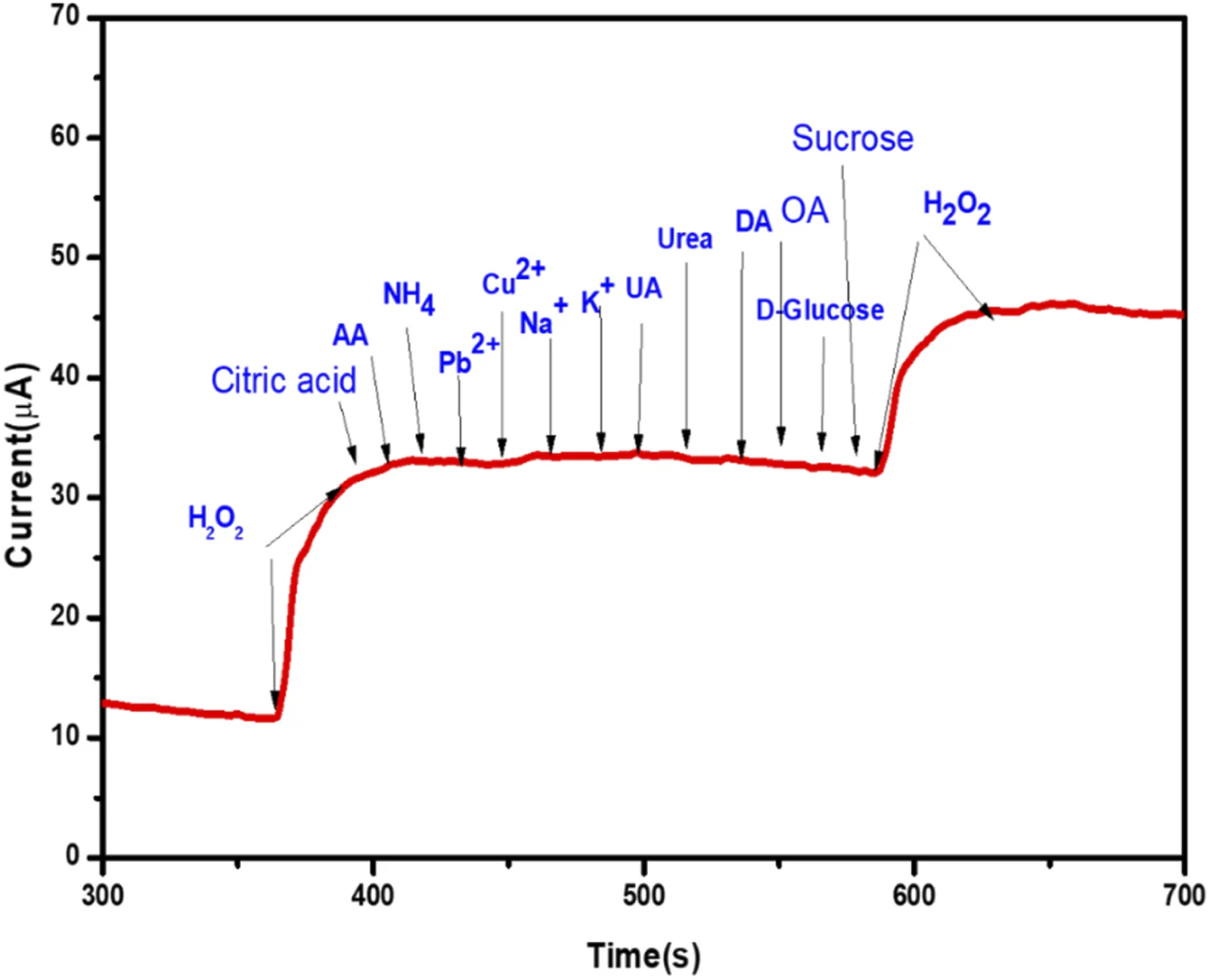
Chronoamperometric response of the MnO_2_–Co_3_O_4_@RGO-modified electrode to 0.1 mM H_2_O_2_ in the presence of different potentially interfering species (0.2 mM each), including ascorbic acid (AA), ammonium chloride (NH_4_Cl), lead nitrate [Pb(NO_3_)_2_], d-glucose, citric acid, sucrose, copper(ii) sulfate pentahydrate (CuSO_4_·5H_2_O), sodium chloride (NaCl), potassium chloride (KCl), uric acid (UA), urea, dopamine (DA), and oleic acid (OA), followed by a final addition of 0.1 mM H_2_O_2_ in PBS (pH 7.4).

### 3.7. Reproducibility, repeatability, and storage stability of the sensor

The reproducibility of the MnO_2_–Co_3_O_4_@RGO-modified electrode was evaluated using five independently prepared electrodes at a fixed H_2_O_2_ concentration of 0.4 mM mM under the optimized chronoamperometric conditions. As shown in Fig. 11A, the electrodes exhibited closely comparable oxidation-current responses, with a relative standard deviation (RSD) of 2.31%. The low inter-electrode variation demonstrates good reproducibility of the electrode fabrication procedure and the resulting analytical response.

**Fig. 11:**
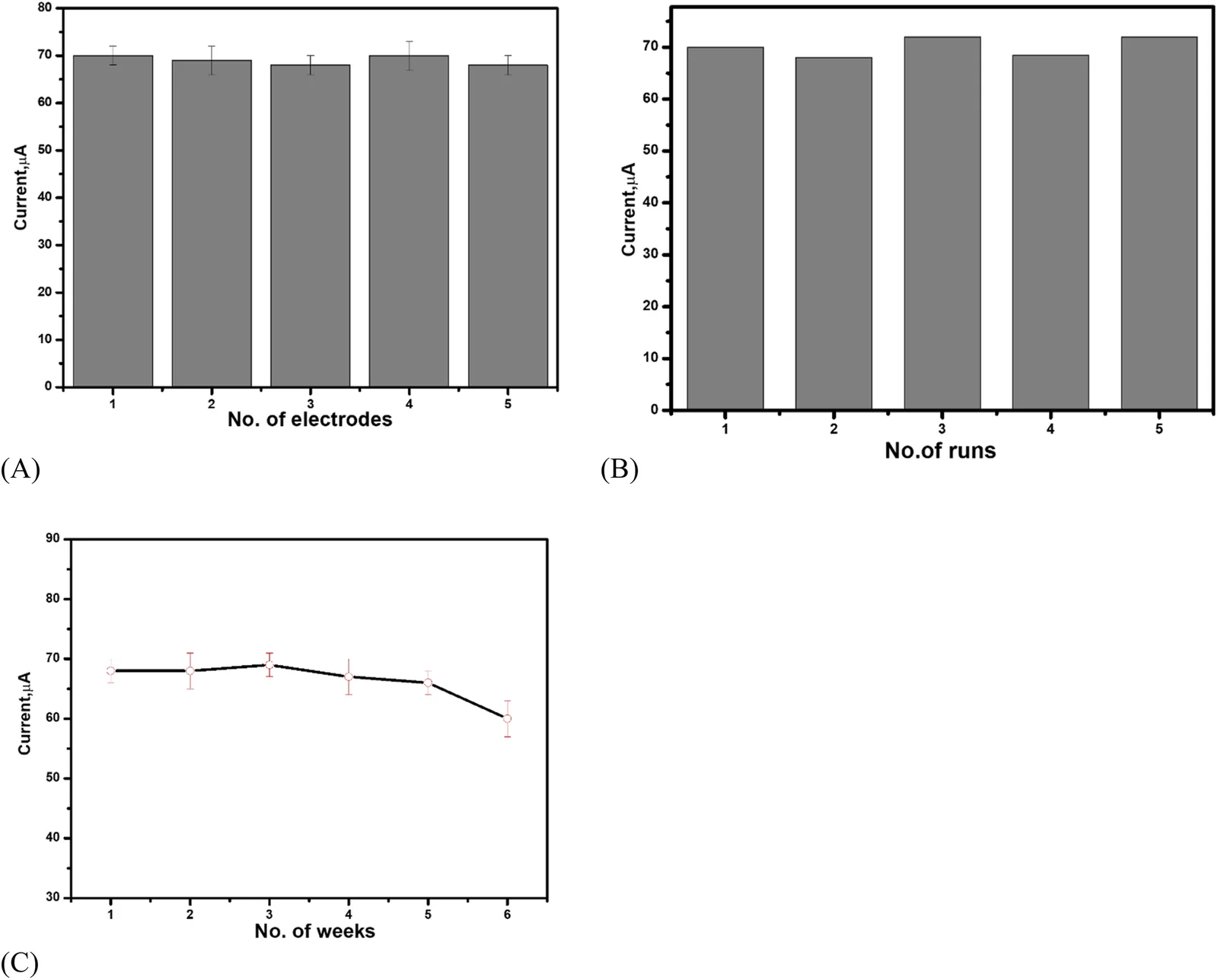
Reproducibility, repeatability, and storage stability of the MnO_2_–Co_3_O_4_@RGO-modified electrode: (A) reproducibility evaluated using five independently prepared electrodes at 0.4 mM H_2_O_2_; (B) repeatability evaluated using a single electrode for repeated measurements of 0.4 mM H_2_O_2_; and (C) storage stability evaluated over six weeks using 0.4 mM H_2_O_2_ under the optimized conditions.

Furthermore, the repeatability of the electrochemical response was evaluated using a single freshly prepared electrode for repeated measurements of 0.4 mM H_2_O_2_ under identical experimental conditions. As shown in Fig. 11B, the repeated measurements exhibited an RSD of only 1.011%, indicating good repeatability of the sensor response under the investigated conditions.

The storage stability of the modified electrode was subsequently evaluated over a period of six weeks. Between measurements, the electrodes were stored in a closed container at ambient laboratory temperature (25 ± 2 °C). As shown in Fig. 11C, the sensor retained approximately 97.8% of its initial response after six weeks, corresponding to a decrease of approximately 2.2% relative to the initial response. The limited change in response during the storage period indicates good storage stability of the MnO_2_–Co_3_O_4_@RGO-modified electrode under the specified storage conditions.

Therefore, the low inter-electrode variability, excellent repeatability of repeated measurements, and retention of 97.8% of the initial response after six weeks demonstrate the consistent analytical performance and favorable storage stability of the proposed sensor.

### 3.8. Peroxide detection in real samples

The developed sensor was designed for practical application in real-sample analysis and exhibited high sensitivity and selectivity toward peroxide detection. Under the optimized conditions, peroxide concentrations were determined in various food and cooked oil samples using the standard addition method (see Table 3). Food or oil samples after sample preparation were analyzed chrono-amperometrically (*n* = 3) using individual modified electrodes. The electrochemical response of each sample was measured before and after spiking with a known concentration of peroxide standard solution, and the peroxide content was calculated from the corresponding change in current signal. As presented in Table 3, the method achieved excellent recoveries ranging from 98.5% to 100.1%, with relative standard deviation (RSD) values between 0.41% and 0.83%, demonstrating the accuracy, precision, and reliability of the proposed sensor for real-sample analysis. Therefore, these results underscore the strong potential of the nanocomposite-based sensor for portable, direct, and on-site peroxide monitoring in food samples. In addition, the sensor offers advantages including simple fabrication, low cost, and user-friendly operation without the need for specialized training.

**Table 3:** Peroxide determination in reals food and oil samples using the newly developed peroxide sensor

Sample	Detected (µM)	H_2_O_2_		Recovery (%) ± RSD
	(A)	Added (µM)	Found (µM)	
				(C)/(A + B)*100
		(B)	(C)	
Chicken salami	10	100	109	99.0 ± 0.51
		400	399	97.3 ± 0.72
		800	798	98.5 ± 0.83
Meat salami	13	100	112	99.1 ± 0.41
		400	413	100 ± 0.68
		800	814	100.1 ± 0.78
Sausage	24	100	123	99.1 ± 0.45
		400	424	100 ± 0.22
		800	825	100.1 ± 0.35
Burger	45	100	145	100.0 ± 0.53
		400	444	99.7 ± 0.68
		800	846	100.1 ± 0.79
Oil (sample 1)	50	100	151	100.6 ± 0.50
		400	450	100.0 ± 0.62
		800	849	99.8 ± 0.79
Oil (sample 2)	74	100	174	99.0 ± 0.61
		400	475	97.3 ± 0.80
		800	875	98.5 ± 0.78

## 4. Conclusion

A non-enzymatic electrochemical sensor based on a MnO_2_–Co_3_O_4_@RGO-modified carbon paste electrode (CPE) was successfully developed for the direct determination of H_2_O_2_. Physicochemical and electrochemical characterization of the investigated electrode materials identified the MnO_2_–Co_3_O_4_@RGO-modified electrode as the most suitable configuration for subsequent H_2_O_2_ sensing studies. The incorporation of RGO provided a conductive carbon framework within the composite, while the MnO_2_ and Co_3_O_4_ phases provided electrochemically active oxide surfaces for H_2_O_2_ conversion. The electrochemical sensing conditions were systematically optimized with respect to the electrolyte pH and applied potential. Under the optimized chronoamperometric conditions, the sensor exhibited a linear response to H_2_O_2_ over the concentration range of 0.1–1800 µM, with a sensitivity of 0.1726 µA µM^−1^, *R*^2^ = 0.996, a calculated LOD of 0.03 µM, and a response time of approximately 12 s. The sensor also demonstrated good reproducibility, with an RSD of 2.31% for five independently prepared electrodes, and good repeatability, with an RSD of 1.011% for repeated measurements using a single electrode. Furthermore, the modified electrode retained approximately 97.8% of its initial response after six weeks of storage at 25 ± 2 °C, indicating good storage stability under the investigated conditions. The interference study showed that the selected food-related compounds, inorganic species, oleic acid, and electroactive compounds produced no appreciable current response under the tested conditions, whereas H_2_O_2_ generated a distinct chronoamperometric response. These results indicate favorable tolerance toward the investigated potential interferents. The successful determination of H_2_O_2_ in real food and edible-oil samples, together with the obtained recovery results, further supports the applicability of the proposed sensor to the analyzed matrices. Therefore, the MnO_2_–Co_3_O_4_@RGO/CPE provides a sensitive, reproducible, and storage-stable platform for non-enzymatic H_2_O_2_ determination. Its broad analytical range, low calculated detection limit, and rapid response demonstrate its potential for H_2_O_2_ analysis in food- and oil-related samples. Future work may focus on integrating the sensing electrode with a compact potentiostat and digital or smartphone-based data acquisition system to facilitate portable measurements outside the laboratory.

## Conflicts of interest

There are no conflicts to declare.

## Data Availability

The authors confirm that the data supporting the findings of this study are available within the article.

## References

[cit1] The global challenge of cancer (2020). Nat. Cancer.

[cit2] Ganesan K., Sukalingam K., Xu B. (2019). Crit. Rev. Food Sci. Nutr..

[cit3] Nambiar V. K., Mudliar V., Salababa I. (2025). Heliyon.

[cit4] Lee J. M., Chang P. S., Lee J. (2007). Korean J. Food Sci. Technol..

[cit5] Bayir H. (2005). Crit. Care Med..

[cit6] Young I. S., Woodside J. V. (2001). J. Clin. Pathol..

[cit7] Ayala A., Muñoz M. F., Argüelles S. (2014). Oxid. Med. Cell. Longevity.

[cit8] Ermak G., Davies K. J. A. (2002). Mol. Immunol..

[cit9] Abbas M. E., Luo W., Zhu L., Zou J., Tang H. (2010). Food Chem..

[cit10] Wu S., Tan N., Lan D., Au C.-T., Yi B. (2020). Int. J. Electrochem. Sci..

[cit11] Sheng Y., Yang H., Wang Y., Han L., Zhao Y., Fan A. (2017). Talanta.

[cit12] Kawser Hossain M., Abdal Dayem A., Han J., Yin Y., Kim K., Kumar Saha S., Yang G.-M., Choi H. Y., Cho S.-G. (2016). Int. J. Mol. Sci..

[cit13] Farrokhnia M., Karimi S., Momeni S., Khalililaghab S. (2017). Sens. Actuators, B.

[cit14] Matsubara C., Kawamoto N., Takamura K. (1992). Analyst.

[cit15] Nakashima K., Wada M., Kuroda N., Akiyama S., Imai K. (1994). J. Liq. Chromatogr..

[cit16] Liu X., Tian H., Yang L., Su Y., Guo M., Song X. (2018). Sens. Actuators, B.

[cit17] Ouda E., Yousf N., Elzwawy A., Magar H. S., Hassan R. Y. A., El-Ashry M., Duraia E.-S. M. (2025). RSC Adv..

[cit18] Mahfouz A. R., Nada H. G., Magar H. S., ElBaghdady K. Z., Hassan R. Y. A. (2025). Int. J. Biol. Macromol..

[cit19] Magar H. S., Abdelghany H., Abbas M. N., Bilitewski U., Hassan R. Y. A. (2023). Microchem. J..

[cit20] Aboarayes D. A., Magar H. S., Hendawy H. A. M., Hassan R. Y. A. (2026). RSC Adv..

[cit21] Magar H. S., El Nahrawy A. M., Hassan R. Y. A., Abou Hammad A. B. (2024). RSC Adv..

[cit22] Magar H. S., Fayez M., Febbraio F., Hassan R. Y. A. (2025). Anal. Biochem..

[cit23] Magar H. S., Hassan R. Y. A., Abbas M. N. (2023). Sci. Rep..

[cit24] Hammad A. B. A., Magar H. S., Mansour A. M., Hassan R. Y. A., Nahrawy A. M. E. (2023). Sci. Rep..

[cit25] Magar H. S., Duraia E.-S. M., Hassan R. Y. A. (2025). Sci. Rep..

[cit26] Ensafi A. A., Jafari-Asl M., Rezaei B. (2013). Talanta.

[cit27] Wang Q., Wang Y., Xiao G., Zhu X. (2023). Materials.

[cit28] Li Y., Tang L., Deng D., He H., Yan X., Wang J., Luo L. (2021). Mater. Sci. Eng. C.

[cit29] Zou M., Feng L., Lin X., Ni Y. (2021). Anal. Methods.

[cit30] Zhao L., Yu X., Zheng S., Zhang K. (2026). Electroanalysis.

[cit31] Kardash-Strochkova E., Tur'yan Y. I., Kuselman I. (2001). Talanta.

[cit32] Ajab H., Jafry A. T., Sajid H., Addicoat M. A., Ayub K., Haq M. Z. U. (2024). Food Chem..

[cit33] Shi F., Geng J., Ara B., Wang B., Li X., Ma Q., Wang L., Fu W., Ahmad M., Sun W. (2024). Microchem. J..

[cit34] Duanghathaipornsuk S., Farrell E. J., Alba-Rubio A. C., Zelenay P., Kim D.-S. (2021). Biosensors.

[cit35] Báez D. F., Brito T. P., Espinoza L. C., Méndez-Torres A. M., Sierpe R., Sierra-Rosales P., Venegas C. J., Yáñez C., Bollo S. (2021). Microchem. J..

[cit36] Magar H. S., Brahman P. K., Hassan R. Y. A. (2022). Biosens. Bioelectron.:X.

[cit37] Magar H. S., Shekhar A., Bilitewski U., Hassan R. Y. A. (2025). Adv. Compos. Hybrid Mater..

[cit38] Karthik R., Sukanya R., Chavan P. R., Kumar D. R., Lee J., Shim J.-J. (2024). Appl. Mater. Today.

[cit39] Wu W., Wu L., Wu H., Wang S., Ding Y., Feng C. (2017). Sens. Actuators, B.

[cit40] Yin D., Bo X., Liu J., Guo L. (2018). Anal. Chim. Acta.

[cit41] Wu W., Li Y., Jin J., Wu H., Wang S., Xia Q. (2016). Sens. Actuators, B.

[cit42] Gao W., Tjiu W. W., Wei J., Liu T. (2014). Talanta.

[cit43] Suresh Babu R., Prabhu P., Sriman Narayanan S. (2013). Talanta.

[cit44] Ali M., Tahir M. N., Siwy Z., Neumann R., Tremel W., Ensinger W. (2011). Anal. Chem..

[cit45] Ma X., Tang K.-l., Yang M., Shi W., Zhao W. (2021). J. Mater. Sci..

[cit46] Zhao D., Xu C. (2015). J. Colloid Interface Sci..

[cit47] Sivakumar M., Veeramani V., Chen S.-M., Madhu R., Liu S.-B. (2019). Microchim. Acta.

[cit48] Noorbakhsh A., Salimi A. (2009). Electrochim. Acta.

[cit49] Achari D. S., Santhosh C., Deivasegamani R., Nivetha R., Bhatnagar A., Jeong S. K., Grace A. N. (2017). Microchim. Acta.

[cit50] Yu Z., Li H., Zhang X., Liu N., Zhang X. (2015). Talanta.

[cit51] Wu X., Li F., Zhao C., Qian X. (2018). Sens. Actuators, B.

[cit52] Lu Z., Wu L., Zhang J., Dai W., Mo G., Ye J. (2019). Mater. Sci. Eng. C.

